# Global prevalence, incidence, and outcomes of alcohol related liver diseases: a systematic review and meta-analysis

**DOI:** 10.1186/s12889-023-15749-x

**Published:** 2023-05-11

**Authors:** Xuanxuan Niu, Lin Zhu, Yifan Xu, Menghan Zhang, Yanxu Hao, Lei Ma, Yan Li, Huichun Xing

**Affiliations:** 1 Center of Liver Diseases Division 3, Beijing Ditan Hospital, Capital Medical University, 8 Jingshundong Street, Chaoyang District, Beijing, 100015 China; 2 Peking University Ditan Teaching Hospital, Beijing, 100015 China

**Keywords:** Alcohol related liver diseases, Epidemiology, Prevalence

## Abstract

**Background:**

Alcohol related liver disease (ARLD) is one of the major chronic liver diseases worldwide. This review aimed to describe the global prevalence, incidence, and outcomes of ARLD.

**Methods:**

Medline, Embase, The Cochrane Library, and China National Knowledge Infrastructure (CNKI) were searched from inception to May 31, 2022. The language was restricted to English or Chinese. According to the criteria, articles describing the basic characteristics of the population were selected. Two reviewers extracted the data independently.

**Results:**

A total of 372 studies were identified: 353 were used for prevalence analysis, 7 were used for incidence analysis, and 114 were used to for outcome analysis. The prevalence of ARLD worldwide was 4.8%. The prevalence in males was 2.9%, which was higher than female (0.5%). Among the ethnic groups, the percentage was highest in Caucasians (68.9%). Alcoholic liver cirrhosis comprised the highest proportion in the disease spectrum of ARLD at 32.9%. The prevalence of ascites in ARLD population was highest (25.1%). The ARLD population who drinking for > 20 years accounted for 54.8%, and the average daily alcohol intake was 146.6 g/d. About 59.5% of ARLD patients were current or former smokers, and 18.7% were complicated with hepatitis virus infection. The incidence was 0.208/1000 person-years. The overall mortality was 23.9%, and the liver-related mortality was 21.6%.

**Conclusion:**

The global prevalence of ARLD was 4.8% and was affected by sex, region, drinking years, and other factors. Therefore, removing the factors causing a high disease prevalence is an urgent requisite.

**Trial registration:**

PROSPERO Nr: CRD42021286192

**Supplementary Information:**

The online version contains supplementary material available at 10.1186/s12889-023-15749-x.

## Background

According to *Global Status Report on Alcohol and Health 2018* [1], about 2.3 billion people are drinking alcohol worldwide currently, and more than half of the population in the USA, Europe, and the Western Pacific consumes alcohol. Chronic heavy drinking is the etiology or risk factor for many diseases, such as alcohol related liver diseases (ARLD)**,** acute pancreatitis, and alcohol-related cardiomyopathy [2]. A study showed a 2.1-fold increase in deaths from alcohol poisoning between 2000 and 2019 in USA [3]. According to the WHO data, the global number of deaths caused by alcohol was about 3 million in 2016. Among them, the deaths caused by alcohol-related digestive diseases accounted for 21.3% of all diseases with highest proportion [1]. The total number of deaths was 637,000, including 607,000 cases of ARLD [1]. Therefore, ARLD has become one of the major causes of alcohol-related death.

Due to the popularity of hepatitis B vaccine and the effective application of antiviral therapy worldwide, the status of the HBV as the main cause of the chronic liver disease is declining gradually [4], while alcohol has gained increasing attention. A study [5] showed that the global prevalence of chronic liver diseases due to alcohol use increased by 3.73% between 2005 and 2015. The prevalence of alcoholic liver cirrhosis in cirrhosis population was increased by 43% in 7 years in the USA [6]. The number of deaths from ARLD in South Korea increased from 1403 to 3588 between 2000 and 2009 [7]. These studies suggested that the burden of ARLD is increasing gradually. Therefore, understanding the epidemiology of ARLD is essential to formulate the relevant prevention and control policies.

However, the current epidemiological data on ARLD were obtained from small-scale research. There is no global consensus. In addition to alcohol consumption as a direct factor of liver injury [8, 9], region, gender [10], race, smoking, and other factors have an impact on the prevalence of ARLD. Thus, this meta-analysis described the characteristics of ARLD population in epidemiology, which could help improve the healthcare strategies and reduce the global prevalence of the disease.

## Methods

This meta-analysis of observational studies was conducted according to the Preferred Reporting Items for Systematic Reviews and Meta-Analyses (PRISMA) and registered in PROSPERO.

### Search strategy and selection criteria

Pubmed, Embase, The Cochrane Library, and CNKI, were searched using the keywords “Liver Diseases, Alcoholic”, “Alcoholic Liver Diseases”, “Alcoholic Liver Disease”, and “Liver Disease, Alcoholic”. The details are described in the Supplementary Table 1. Studies published from the respective inception dates of the databases to May 31, 2022 were eligible for inclusion in this meta-analysis. The language of the literature was limited to English or Chinese.

### Data extraction and quality assessment

Two reviewers searched the studies and extracted the data independently. Any disagreements on the eligible studies and data extraction were resolved by consensus and/or discussion with a third author. The included literature is listed in the Supplementary Tables 2, 3, 4 and 5. Newcastle–Ottawa scale was used to evaluate the quality of the studies, ranging from 0–9: 7–9 represented a high-quality score, 4–6 represented a medium score, and 1–3 represented a low score. Studies with scores < 4 were excluded.

### Study definitions

ARLD is a series of liver injuries caused by long-term high-alcohol intake, including mild alcoholic liver disease, alcoholic fatty liver disease, alcoholic hepatitis, alcoholic cirrhosis, and related complications [11]. ARLD was diagnosed according to long-term drinking history or short-term heavy drinking history, without autoimmune hepatitis, drug-induced liver diseases, or other genetic disorder related liver diseases. It should be evaluated though blood biochemical testing, ultrasound, transient elasticity, CT, MRI, and biopsy [12, 13]. The concrete situation was determined based on the latest diagnostic criteria of the corresponding year. The general population without defined diseases was referred to the physical examination at the health care center and participated in the epidemiological survey. Original research articles that defined their population as ARLD and/or general population were included. Including studies should also provide the data on the prevalence, incidence and outcomes of ARLD. Study designs with unrestricted types were eligible for inclusion. In case of duplicate data, the largest and latest datasets were selected. Articles were excluded if the diagnosis of ARLD were unclear; the number of participations were < 50 in the baseline; age < 18-years-old; the study population was limited to either one gender; specific groups with other chronic diseases, such as non-alcoholic liver disease, drug-induced liver disease, and acquired immune deficiency syndrome.

### Data analysis

The incidence of ARLD was studied in the general population at baseline without the disease. To estimate the incidence, we used the number of new cases and the follow-up time (person-years). The baseline characteristics of ARLD population, including prevalence, mortality, and cause of death, were described. In order to study the influencing factors of prevalence, we analyzed region, sex, race, disease severity, complications, drinking years, smoking, virus infection, and other factors. Moreover, the global prevalence was compared between 2000–2010 and 2011–2021.

### Statistical analysis

Cochran Q and *I*^2^ statistics were used to assess the heterogeneity. *p*-value < 0.05 in Q-statistic and *I*^2^ ≥ 50% were considered moderate or severe heterogeneity. Due to the heterogeneity of global data, random-effects model was applied to analyze each study dataset. Funnel plot and Egger’s test (the figures of main result are listed in Supplementary Figs. 1– 5) were used to evaluate the publication bias. All statistical analyses were conducted using Meta package in Stata statistical software.

## Results

### Study selection and characteristics

**Fig. 1:**
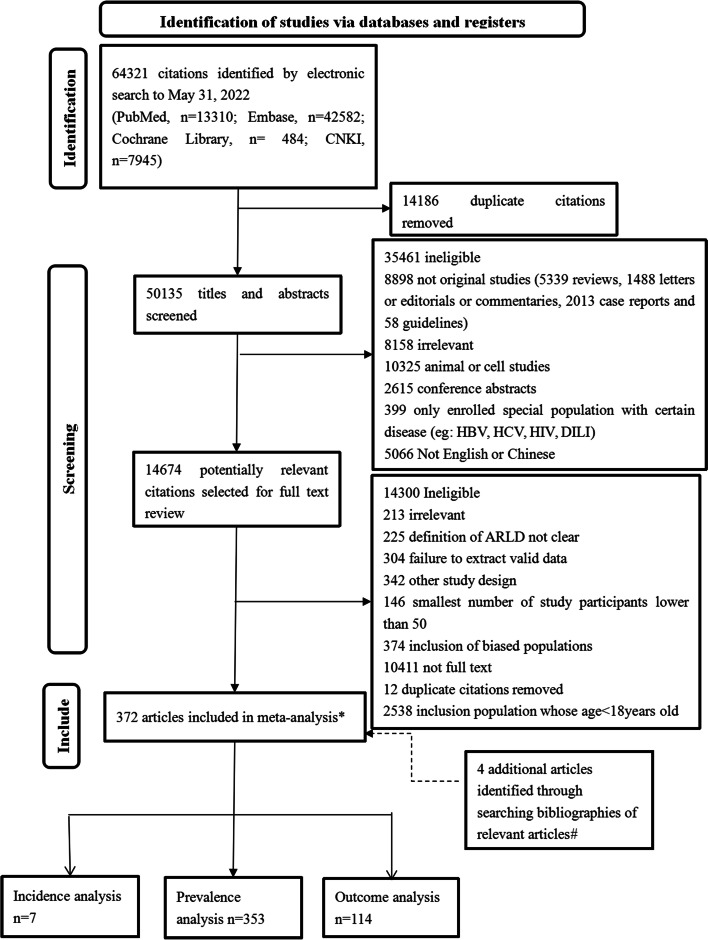
Study selection. The entire screening process were described, including the selected the reasons for the exclusion of articles and the number of including and excluding artcles. ARLD: alcohol-associated liver disease. HBV: hepatitis B virus. HCV: hepatitis C virus. HIV: human immunodeficiency virus. DILI: drug-induced liver injury.* Some articles were used for more than one of the analyses of prevalence, incidence, and outcomes. # When searching for relevant articles found 4 additional articles that met the inclusion criteria

According to the above-defined search terms in methods, 64,321 studies were retrieved, and 368 studies were included according to the inclusion criteria. While searching for relevant articles, four additional studies were added. Finally, 372 articles were included: 353 studies for prevalence analysis, 7 for incidence analysis, and 114 for outcome analysis (some studies provided data on prevalence, incidence and/or outcomes at the same time; hence, the total number was different from the sum of subgroups) (Fig. 1).

### Prevalence of ARLD

The overall prevalence of ARLD in general population was 4.8% [95% confidence interval (CI): 4.1–5.6] (99 studies, 198,423,289 participations) (Table 1). The prevalence fluctuated from 1.0 to 16.1% and higher in male compared to female (2.9% *vs*. 0.5%, *p* < 0.001).

**Table 1:** The prevalence of ARLD

	**Studies (N)**	**Participations (N)**	**ARLD (n)**	**Prevalence (%, 95%CI)**	***I***^2^**	***P***
***By country area***						
Overall	99	198,423,289	1,806,506	4.8% (4.1–5.6)	99.998	< 0.001
Portugal	1	773,187	7751	1.0% (0.9–1.1)	-	< 0.001
Canada	1	690,401	7112	1.0% (1.0–1.1)	-	< 0.001
Iceland	1	16,000	317	1.0% (0.8–1.2)	-	< 0.001
Denmark	3	5,531,764	11,260	1.2% (0.1–3.4)	99.990	0.046
France	2	261,248	3663	1.4% (1.3–1.4)	-	< 0.001
South Korea	4	48,933,410	41,262	2.3% (0.1–7.7)	99.934	< 0.001
China	54	2,976,478	29,681	3.9% (2.9–5.1)	99.902	< 0.001
USA	17	137,929,285	1,664,682	5.0% (2.9–7.6)	100	< 0.001
UK	5	1,187,539	23,457	7.2% (3.0–13.0)	99.989	< 0.001
Japan	6	88,043	12,318	10.4% (3.2–20.9)	99.931	< 0.001
Uganda	1	8099	888	11.0% (7.7–10.2)		< 0.001
India	2	3229	422	11.8% (10.7–12.9)	-	< 0.001
Sweden	1	4611	645	14.0% (13.0–15.0)	-	< 0.001
Italy	3	19,995	3206	16.1% (1.2–43.3)	-	0.009
***By regions***						
Asia	66	52,001,160	83,683	4.5% (4.0–5.5)	99.950	< 0.001
North America	17	138,619,686	1,671,794	4.7% (2.7–7.1)	100	< 0.001
Europe	16	7,794,344	50,141	5.4% (3.9–7.1)	99.980	< 0.001
***Province of China***^**a**^						
Overall	54	2,976,478	29,681	3.9% (2.9–5.1)	99.903	< 0.001
Sichuan	5	27,775	652	1.8% (1.1–2.6)	94.661	< 0.001
Beijing	4	2,660,342	16,051	1.9% (0.1–5.5)	99.989	0.007
Guangdong	5	27,835	878	1.9% (0.7–3.6)	98.563	0.001
Jiangsu	2	8453	190	2.2% (1.9–2.5)	-	< 0.001
Shanghai	2	6192	192	2.9% (2.5–3.3)	-	< 0.001
Gansu	2	50,342	1627	3.2% (3.1–3.4)	-	< 0.001
Shaanxi	3	16,572	652	3.5% (1.7–6.0)	-	< 0.001
Guizhou	2	20,229	776	3.8% (3.6–4.1)	-	< 0.001
Zhejiang	7	37,375	1904	4.2% (1.8–7.5)	99.410	< 0.001
Henan	2	5648	241	4.2% (3.7–4.7)	-	< 0.001
Hunan	2	19,696	836	4.2% (3.9–4.5)	-	< 0.001
Jilin	4	14,462	756	4.7% (3.1–6.7)	95.180	< 0.001
Heilongjiang	1	1203	58	4.8% (3.7–6.2)	-	< 0.001
Taiwan	1	46,565	2249	4.8% (4.6–5.0)	-	< 0.001
Tibet	1	2178	106	4.9% (4.0–5.9)	-	< 0.001
Liaoning	1	7420	368	5.0% (4.5–5.5)	-	< 0.001
Yunnan	2	2190	159	6.8% (5.8–7.9)	-	< 0.001
Anhui	2	6273	581	8.0% (7.3–8.7)	-	< 0.001
Shandong	2	8495	692	8.1% (7.5–8.7)	-	< 0.001
Hebei	1	866	85	9.8% (7.9–12.0)	-	< 0.001
Xinjiang	1	2567	274	10.7% (9.5–11.9)		< 0.001
Northeast China^b^	5	22,851	1171	4.8% (3.7–6.0)	98.617	< 0.001
Northwest China^c^	8	73,281	2907	5.9% (4.2–7.9)	99.739	< 0.001
***By sex***	58	195,227,050	1,265,327		99.993	
Male			924,436	2.9% (2.4–3.5)		< 0.001
Female			340,891	0.5% (0.4–0.7)		< 0.001
***By study period***	67	122,004,134	598,920			
2000–2010	49	119,422,376	509,074	4.6% (4.2–5.0)	99.987	< 0.001
2011–2021	18	2,581,758	89,846	5.6% (2.4–10.1)	99.993	< 0.001

Because some articles provide more than one characteristics of population with ARLD, they can be used for multiple subgroup analysis. Therefore, the sum of the articles and population data is not equal to the total

*ARLD* Alcohol related liver disease

^**^All* p* values for* I*^*2*^ are lower than 0.05

^a^Study province referred to study province, Municipality, Autonomous Region, or Special Administrative Region

^b^Northeast China comprised data from Heilongjiang, Jilin, Liaoning

^c^Northwest China comprised data from Xinjiang, Shaanxi, Gansu

### By geographic region and province

**Fig. 2:**
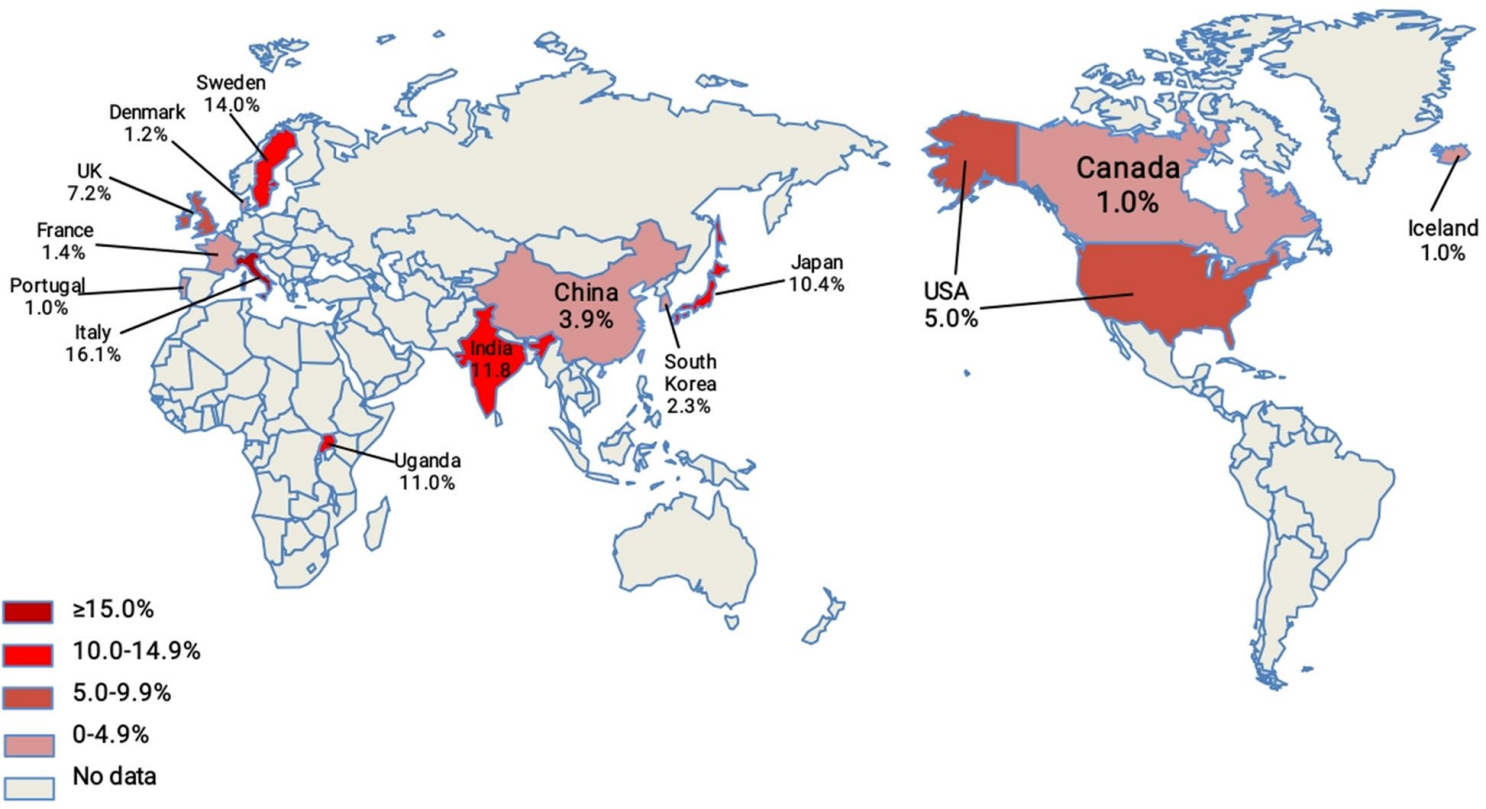
The global prevalence of ARLD. The prevalence in 14 countries was indicated by depth of red. The 14 countries included Portugal, Canada, Iceland, France, China, USA, Denmark, South Korea, Uganda, India, UK, Sweden, Japan, and Italy

**Fig. 3:**
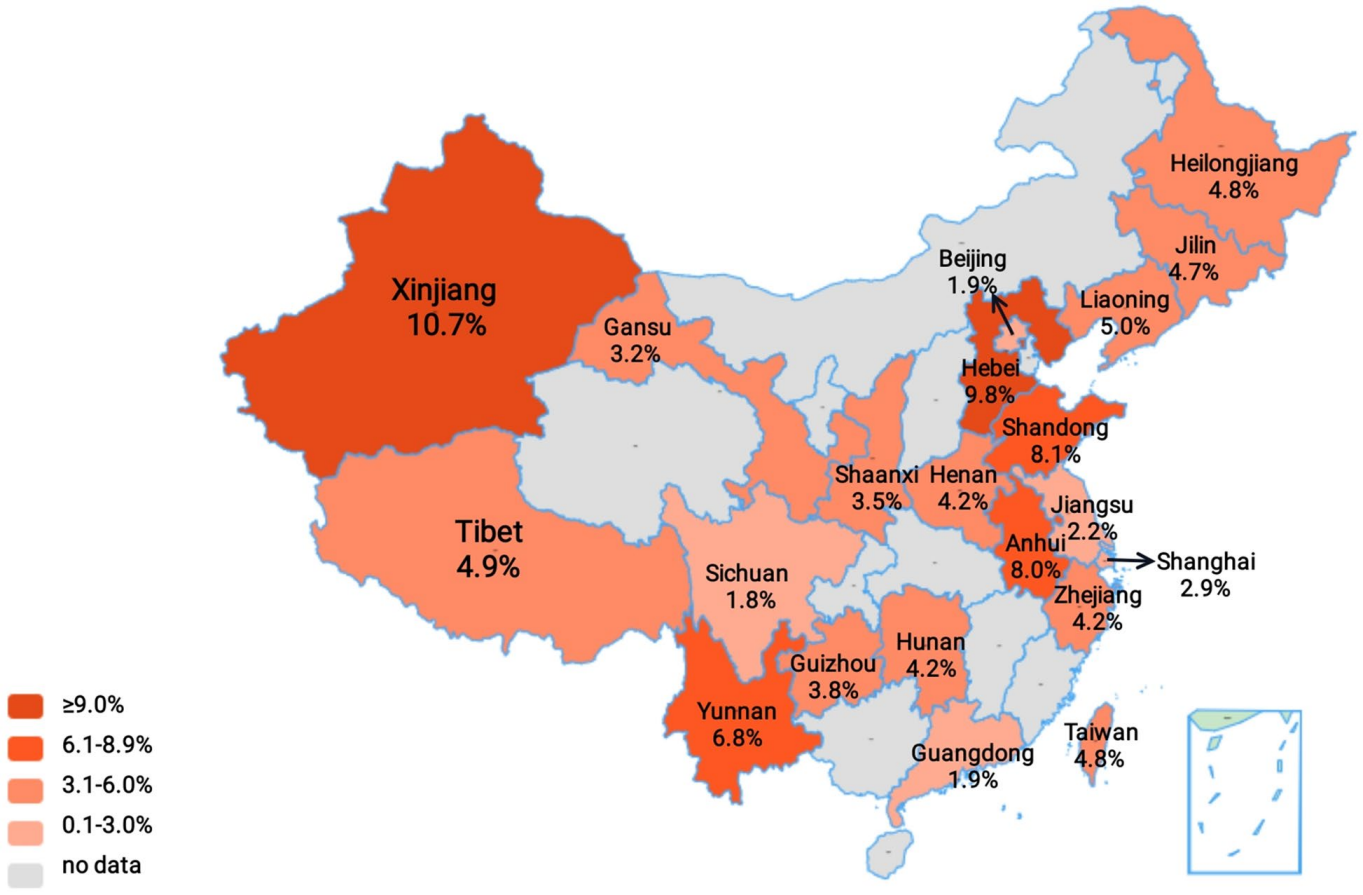
The prevalence of ARLD in China by provinces. The prevalence in 21 provinces of China was indicated by depth of red. The prevalence in China was obtained from the analysis of 21 cities or provinces including Sichuan, Beijing, Guangdong, Jiangsu, Shanghai, Gansu, Shaanxi, Guizhou, Zhejiang, Henan, Hunan, Jilin, Heilongjiang, Taiwan, Tibet, Liaoning, Yunnan, Anhui, Shandong, Hebei, and Xinjiang provinces

The information on the prevalence of ARLD in different countries and regions is summarized in Table 1 and Figs. 2 and 3. The study encompassed 14 countries, including Portugal [14], Canada [15], Iceland [16], France [17, 18], China (see the following paragraph), USA [19–35], Denmark [36–38], South Korea [39, 40], Uganda [41], India [42, 43], UK [10, 44–47], Sweden [48], Japan [49–54], and Italy [55–57](Fig. 2 and Table 1). The global prevalence of ARLD in general population was 4.8% (95% CI: 4.1–5.6) (Fig. 2), which we got from 99 datasets. As the region with the highest alcohol consumption in the world [12], Europe had the highest prevalence (5.4%, 95% CI: 3.9–7.1). According to the analysis results in Table 1 and the color depth distribution in Fig. 2, the prevalence of ARLD was higher in most European countries. Italy had the highest prevalence rate of 16.1% (95% CI: 1.2–43.3), followed by Sweden (14.0%, 95% CI: 13.0–15.0) and the UK (7.2%, 95% CI: 3.0–13.0), while France, Denmark, Iceland and Portugal had the lowest prevalence rates: 1.4% (95% CI: 1.3–1.4), 1.2%(95% CI: 0.1–3.4), 1.0% (95% CI: 0.8–1.2) and 1.0% (95% CI: 0.9–1.1), respectively (Table 1, Fig. 2). Although alcohol consumption in Asia was less than that in Europe, the prevalence in India (11.8%, 95% CI: 10.7–12.9) and Japan (10.4%, 95% CI: 3.2–20.9) was high (Table 1). Uganda in Africa has the high prevalence of 11.0% (95% CI: 7.7–10.2). Surprisingly, the United States, with the largest number of studies (17 studies) and participants (137,929,285 persons), had the lower prevalence (5.0%, 95% CI: 2.9–7.6) (Table 1).

Since the pathogenesis of ARLD in Western countries, which has been well-documented, differed from that in China [9], the prevalence in China’s provinces was analyzed separately. The prevalence of ARLD was 3.9% (95% CI: 2.9–5.1) (Table 1) in Chinese people, which was lower than the global prevalence. Data from 21 cities or provinces, including Sichuan [58–62], Beijing [63–66], Guangdong [67–71], Jiangsu [72, 73], Shanghai [74, 75], Gansu [76, 77], Shaanxi [78–80], Guizhou [81, 82], Zhejiang [83–89], Henan [90, 91], Hunan [92, 93], Jilin [94–97], Heilongjiang [98], Taiwan [99], Tibet [100], Liaoning [101], Yunnan [102, 103], Anhui [104, 105], Shandong [106, 107], Hebei [108], and Xinjiang [109] provinces, were collected (Fig. 3 and Table 1). The prevalence in Xinjiang was 10.7% (95% CI: 9.5–11.9), which was much higher than the overall prevalence in China. It was the province with the highest prevalence, followed by Hebei (9.8%, 95% CI: 7.9–12.0) and Shandong provinces (8.1%, 95% CI 7.5–8.7), which were famous for strong drinking. Surprisingly, Shaanxi and Gansu provinces, famous for binge drinking, had the lower prevalence. Further analysis of Northeast China [95–98, 101] (including Heilong, Jilin, and Liaoning provinces) and Northwest China [76–80, 109–111] (including Xinjiang, Shaanxi, and Gansu provinces) showed that the prevalence was 4.8% (95% CI: 3.7–6.0) and 5.9% (95% CI: 4.2–7.9), which were much higher than the national prevalence. The prevalence rates were relatively low in Sichuan (1.8%, 95% CI: 1.1–2.6), Beijing (1.9%, 95% CI: 0.1–5.5), Guangdong (1.9%, 95% CI: 0.7–3.6), Jiangsu (2.2%, 95% CI: 1.9–2.5), and Shanghai (2.9%, 95% CI: 2.5–3.3), all which were relatively economically developed regions (Table 1).

### By sex

Herein, 58 studies [14–16, 18–20, 22–25, 29–31, 33–40, 44–46, 50, 53, 55, 56, 58–60, 63–67, 71, 77, 78, 80, 81, 84–86, 92, 93, 96, 97, 99–101, 104–107, 111] were included to analyze the influence of sex on the prevalence of ARLD. The prevalence of male was 2.9% (95% CI: 2.4–3.5), which was much higher than that of women (0.5%, 95% CI: 0.4–0.7) (Table 1).

### By study period

In order to further analyze the changes in the global ARLD prevalence in different periods, seventy three articles indicating the study years were included. From these, 48 articles [10, 20, 25, 26, 30, 34, 35, 37, 40, 42–44, 48, 50, 53, 59–63, 67–69, 71–74, 76, 80, 81, 83, 85, 87–91, 93–97, 99–102, 109, 111] were used for subgroup analysis of the prevalence from 2000 to 2010: 4.6% (95% CI: 4.2–5.1) (Table 1). A total of 18 articles [10, 17, 19, 22, 26, 41, 43, 49, 51, 52, 65, 70, 75, 78, 79, 82, 104, 106] were used to analyze the prevalence in 2011–2021, and the result was 5.6% (95% CI: 2.4–10.1), which was significantly higher in this period than in 2000–2010 (Table 1).

### The characteristic of ARLD

The characteristics of ARLD patients in nationality, race, disease severity, complication, drinking years, smoking/smoked, and hepatitis virus infection are different. This part was described by comparing the percentage of each part of ARLD population.

### By race and nationality

For race, categories included Caucasians, Africans, Hispanic and Asians, and these are the relatively lager amount of data that we can gather from the studies. According to the 46 articles [20–26, 29–31, 33, 35, 112–145] collected, the proportion of Caucasians was the highest (68.9%, 95% CI: 67.6–70.2) in ARLD, which was much higher than Africans (8.9%, 95% CI: 8.5–9.4) and Hispanic (8.6%, 95% CI: 7.9–9.4) (Table 2). The Asians was the lowest (0.3%, 95 CI: 0.1–0.6), with wide variation between races.

**Table 2:** The influence of various influencing factors on the prevalence of ARLD

	**Studies (N)**	**ARLD (n)**	**Characteristic (%, 95%CI)**	***I***^2^**	***P***
***Race***	46	4,828,061			
Caucasians		3,146,877	68.9% (67.6–70.2)	99.838	< 0.001
Africans		484,170	8.9% (8.5–9.4)	99.243	< 0.001
Hispanic		814,203	8.6% (7.9–9.4)	99.830	< 0.001
Asians		23,931	0.3% (0.1–0.6)	99.905	< 0.001
***Nationality***	10	2078			
Minorities^a^		1299	61.6% (52.8–70.0)	93.630	< 0.001
Han		779	38.4% (30.0–47.2)	93.630	< 0.001
***Severity of disease***	147	564,855			
Mild Alcoholic liver disease		9730	2.1% (1.3–3.0)	99.675	< 0.001
Alcoholic fatty liver disease		12,566	19.1% (17.0–21.4)	99.695	< 0.001
Alcoholic hepatitis		35,819	16.4% (13.4–19.6)	99.870	< 0.001
Alcoholic cirrhosis		348,848	32.9% (27.3–38.7)	99.939	< 0.001
Other		157,892	6.0% (3.0–9.8)	99.955	< 0.001
***By complication***	119	3,527,134			
SBP		97,135	0.2% (0–0.5)	99.918	0.020
Hepatorenal syndrome		193,512	0.7% (0.4–1.1)	99.820	< 0.001
Infection		119,448	2.4% (1.6–3.3)	99.935	< 0.001
Gastrointestinal haemorrhage		575,413	7.5% (6.0–9.2)	99.951	< 0.001
Encephalopathy		1,006,363	10.6% (8.8–12.6)	99.954	< 0.001
Ascites		1,465,780	25.1% (20.5–30.0)	99.986	< 0.001
***Duration of alcohol intake(yr)***	19	4576			< 0.001
5–9(yr)		449	13.0% (9.1–17.5)	94.043	< 0.001
10–19(yr)		1187	29.5% (24.9–34.3)	90.974	< 0.001
≥ 20(yr)		2940	54.8% (46.9–62.6)	96.366	< 0.001
***Daily dose of pure alcohol consumed (g/d)***	20	4184	146.6 (123.8,169.4)	100	< 0.001
***Smoking***	27	106,599	59.5% (55.9–63.1)	98.779	< 0.001
***By viral infection***	67	1,473,951			< 0.001
HBV		11,712	3.6% (3.0–4.3)	99.637	< 0.001
HCV		66,744	5.6% (4.1–7.4)	99.932	< 0.001
HBV and/or HCV		92,548	18.7% (16.0–21.5)	99.929	< 0.001

Because some articles provide more than one characteristics of population with ARLD, they can be used for multiple subgroup analysis. Therefore, the sum of the articles and population data is not equal to the total

*ARLD* Alcohol related liver disease, *SBP* Spontaneous bacterial peritonitis

^**^All* p* values for* I*^*2*^ are lower than 0.05

^a^The minorities group included Mongol, Chosen, Li, Hmong, Kazak, Uyghurs, Xibe, Hani, Yi and Dai

China has multiple ethnic groups. The data [103, 146–154] collected in this study divided Chinese people with ARLD into Han nationality and other minorities, which including Mongol, Chosen, Li, Hmong, Kazak, Uyghurs, Xibe, Hani, Yi and Dai (Table 2). The minority group accounted for 61.6% (95% CI: 52.8–70.0), which was much higher than in the Han group (38.4%, 95% CI: 30.0–47.2).

### By duration of alcohol intake and the daily dose of pure alcohol consumption

Alcohol as a pathogenic factor, drinking years and average daily drinking consumption affected the natural course of ARLD. According to the data of ARLD population, they were divided into three subgroups with drinking years of 5–9, 10–19, > 20. The ARLD with drinking duration > 20 years accounted for 54.8% (95% CI: 46.9–62.6) (Table 2). The analysis of the 19 studies [78, 80, 81, 84, 92, 95, 146, 148, 149, 151, 155–163] describing the correlation between drinking years and ARLD revealed that the longer the drinking years, the more patients with ARLD. The average daily alcohol consumption of 4184 ARLD patients [98, 133, 140, 153, 164–179] included in the study was up to 146.6 g/d (95% CI: 123.8–169.4) (Table 2), which was much higher than the excessive drinking defined by the National Institute on Alcohol Abuse and Alcoholism (NIAAA): > 4 cups/day for men and > 3 cups/day for women (1 standard cup = 14 g alcohol) [180].

### By smoking and hepatitis viral infectious

As a risk factor of many diseases, tobacco has been proven to increase the risk of liver fibrosis [181]. Among 27 articles [19, 23, 24, 33, 37, 40, 49, 163, 171–173, 179, 182–196], 106,599 people were diagnosed with ARLD, of which 53,661 were former or current smokers, accounting for 59.5% (95% CI: 55.9–63.1) (Table 2). Three articles [37, 186, 188] described that smoking promoted disease progression, and two studies [23, 33] showed that smoking increased the mortality and hospitalization rates. In addition to advising the patients to quit alcohol, education on smoking cessation was also conducive to improving the progress of ARLD.

Hepatitis virus infection and alcohol are the major causes of chronic liver disease. Among the 67 articles [19, 21, 25, 26, 30, 40, 53, 87, 99, 114, 115, 120, 121, 123, 125, 126, 156, 158, 161, 167, 168, 171, 172, 176, 192, 194, 196–236] included, 92,548/1473951 patients with ARLD were complicated with a hepatitis viral infection, with the prevalence of 18.7% (95% CI: 16.0–21.5), and the infection rate of HCV (5.6%, 95% CI: 4.1–7.4) was higher than that of HBV (3.6%, 95% CI: 3.0–4.3) (Table 2). Three of the 67 articles [210, 235, 236] described that the liver damage caused by concurrent hepatitis virus infection and alcohol was severe. One article [194] suggested that the presence of both causes an increased possibility of 30-day readmission. The data of 4 studies [30, 126, 201, 204] showed that the mortality of ARLD with virus infection was higher than that of ARLD only. Therefore, antiviral drugs are essential for ARLD with hepatitis viral infection while abstaining from alcohol.

### By the stage of disease and complications

ARLD caused by long-term heavy drinking included the whole disease spectrum from liver steatosis to liver cirrhosis and even liver cancer [237]. The constituent ratio of alcoholic cirrhosis in ARLD population was the highest, up to 32.9% (95% CI: 27.3–38.7) (Table 2) got from 147 articles [16, 19, 25–27, 31, 36, 46, 64, 66, 80, 81, 84, 86, 91, 92, 98, 100–102, 111, 113, 117, 119, 121–124, 145, 148, 149, 152, 153, 157, 159–162, 165, 170, 173–176, 187, 193, 194, 197, 199, 208, 219, 221–223, 225, 227, 229–234, 236, 238–321]. A total of 119 datasets [25, 26, 30, 43, 47, 113, 115, 116, 122, 125, 126, 128, 129, 131, 132, 136, 137, 142, 153, 156, 157, 161, 162, 168, 171–173, 176, 178, 186, 190, 193–197, 204, 207, 208, 213, 215, 221, 225–230, 241–243, 245, 250–252, 255, 262, 267, 272, 275, 276, 294, 297, 299, 306, 311, 318, 321–372] were used to analyze the prevalence of ascites, gastrointestinal bleeding, hepatic encephalopathy, spontaneous peritonitis (SBP), hepatorenal syndrome, and bacterial infection in ARLD. Ascites were the most common complication, with a prevalence of 25.1% (95% CI: 20.5–30.0), three times that of gastrointestinal bleeding (Table 2).

SBP is the most common and life-threatening bacterial infection in cirrhotic patients with ascites. The prevalence of SBP in ARLD population was not high (0.2%, 95% CI: 0–0.5) (Table 2). However, the prevalence of SBP in alcoholic cirrhosis [25, 122, 132, 194, 204, 208, 356, 362, 368, 373] with ascites was 12.5% (95% CI: 10.7–14.4). Since the clinical symptoms of SBP were often occult and only limited research data were available, the prevalence may be underestimated. The occurrence of the above complications often indicated decompensated alcoholic cirrhosis, which was the predictor of mortality. Diabetes is also one of the critical factors affecting prognosis. A total of 40 studies [26, 33, 77, 131, 145, 156, 159, 171, 172, 176, 178, 183, 207, 215, 220, 221, 223, 226, 228, 260, 270, 294, 295, 297, 306, 307, 348, 360, 368, 372, 374–383] showed that 15.6% (95% CI: 12.8–18.7) ARLD were complicated by diabetes.

### Incidence of ARLD

To estimate the incidence of ARLD, data from 7 eligible cohort studies [16, 39, 47, 384–387] were selected, with a cumulative follow-up of 368,565,116.7 person-years. The number of newly defined ARLD cases was 62,819, and the incidence was 0.208 (95% CI: 0.125–0.305) per 1000 person-years (Table 3). The incidence in males was about four times as high as that in females (0.163 *vs*. 0.035 per 1000 person-years,* p* < 0.05) (Table 3), suggesting a significant gender difference in the incidence.

**Table 3:** The incidence of ARLD in general population

	**Studies (N)**	**General population at baseline (N)**	**ARLD patients (n)**	**Total follow-up (person- years)**	**Incidence (per 1000 person-years, 95%CI)**	***I***^***2***^ ** (%)	***p***
***Incidence***	7	60,619,863	62,819	368,565,116.7	0.208 (0.125–0.305)	100	< 0.001
***Male***			52,048		0.163 (0.095–0.244)	100	< 0.001
***Female***			10,771		0.035 (0.015–0.062)	99.99	< 0.001

*ARLD* Alcohol related liver disease

^**^All* p* values for* I*^*2*^ are lower than 0.05

### Mortality and cause of ARLD death

A total of 114 datasets [17, 23, 25, 26, 30, 33, 36, 39, 43, 46, 64, 115, 126, 128, 130, 136, 140, 142, 156, 162, 168, 169, 173, 176, 186, 189, 190, 192, 196, 197, 204, 208, 213, 216, 220, 223, 225, 228, 231, 236, 242–245, 249, 258, 265, 267, 273, 276, 280, 283, 294, 307, 309, 311, 320–322, 324–329, 332–336, 338–345, 347, 348, 353, 355, 356, 359, 364, 366–375, 380, 382, 385, 386, 388–402] were used to analyze the mortality, including 786,199 ARLD patients, while 183,929 people died during the study (Table 4). The mortality was 23.9% (95% CI: 18.9–29.2). Since not all studies provided the analysis of the causes of death, 22 articles [33, 46, 47, 162, 168, 189, 243, 245, 249, 265, 273, 326, 338, 340, 344, 345, 356, 364, 378, 390, 396, 401] were selected for subsequent analysis, with the mortality of 37.1% (95% CI: 27.7–47.1). According to the funnel plot and Egger’s test, *p* = 0.612 indicated that the funnel plot was symmetrical, i.e., no publication bias in the 22 datasets. A total of 15,965 individuals suffered from ARLD, of which 4746 died of liver disease and related complications. The mortality related to liver diseases was 21.6% (95% CI: 15.8–28.1), about twice that of non-liver diseases (10.4%, 95%CI: 6.3–15.3) (Table 4).

**Table 4:** The outcomes of ARLD

	**Studies (N)**	**Death (n)**	**Mortality (%, 95%CI)**	***I***^2^** ***(%)***	***P***
***Mortality***	114	183,929	23.9% (18.9–29.2)	99.959	< 0.001
***The result of death***	22	8032			< 0.001
Liver-related		4746	21.6% (15.8–28.1)	98.631	< 0.001
Non-liver-related		3053	10.4% (6.3–15.3)	98.602	< 0.001

^**^All* p* values for* I*^*2*^ are lower than 0.05

## Discussion

ARLD is a common chronic liver disease caused by long-term heavy drinking. Alcoholic fatty liver could develop into alcoholic hepatitis characterized by inflammation, which further progresses to alcoholic liver fibrosis, alcoholic cirrhosis, and even cancer in some cases. The whole process is affected by the interaction of many risk factors involved in this article, such as sex, race, hepatitis virus infection and smoking. Genetic and other potential etiology of liver diseases which identified in this article could also affect the natural course of the disease. The co-existence of multiple risk factors could largely promote the progression of ARLD through complex molecular mechanisms. Several studies have shown that alcohol abuse is accompanied by metabolic syndrome [252, 403] or hepatitis virus infection [404, 405], would accelerate the speed of liver fibrosis. Another study demonstrated that 70% of HCV-infected patients in Europe and North America are heavy drinkers [406], which is in agreement with the high prevalence shown in the present study.

According to the *2018 National Survey on Drug Use and Health*, 14.4 million adults ≥ 18-years-old suffered from alcohol use disorders in the United States, including 9.2 million men and 5.3 million women [407]. Since 2000, men have been drinking about three times as much as women, according to the latest WHO data [408]. The current data also showed that the prevalence of ARLD in male was 2.9% which was nearly six times that in female, which could be related to the socioeconomic status and alcohol consumption level of men and women. Although the prevalence of different sexes varied greatly, women’s susceptibility to alcohol cannot be ignored. According to the WHO statistics, the global alcohol consumption is increasing [1], with the average level of alcohol consumption in 2005 was 5.5 L of pure alcohol per capita, and in 2016 was 6.1L [1, 408]. The current findings showed that the prevalence during 2011–2021 was higher than that during 2000–2010, which was consistent with the epidemiological characteristics of alcohol.

Significant differences in alcohol consumption patterns, metabolism, genetics, and socio-economic factors among different subgroups affected the prevalence of ARLD. A direct correlation was established between alcohol and liver disease. The longer the drinking years and/or the higher the average daily drinking amount, the higher the risk of ARLD. Therefore, the distribution of high prevalence rates was consistent with regions where drinking culture was in vogue. Although Europe is still the region with the highest prevalence in the world, Portugal and France are the lowest which have a long history of wine. Thus, it could be deduced that the type of alcohol may also have an impact on the prevalence. Askgaard et al. demonstrated that red wine had a lower risk than other types of alcoholic beverages when drinking the same amount of alcohol [384]. In Uganda, the prevalence of homemade alcoholic beverages is high [409]. Similar issues may be seen in some provinces in China, such as Xinjiang, Hunan and Henan provinces [410]. This phenomenon may be also related to the cultural customs of different ethnic groups. Many minorities had the tradition of homemade alcoholic beverages. For example, Duihua wine, the special homemade wine unique to the Dai nationality, has a high alcohol content of over 60%. Its health risk may also be attributed to its toxic impurities, such as heavy metals and acetaldehyde [411]. The prevalence in USA which is lower than our expectations may be attributed to the government restriction on alcohol consumption. The United States established NIAAA in 1971 [412], and has a stable government funding program. For example, the raised alcohol taxes and the restriction of the time and place of alcohol sales have significantly reduced the incidence of ARLD. The comparison of prevalence in different countries reflected the advantages, which could be used as a reference for countries with a high prevalence. With the improvement of the economic level of developing countries, the prevalence in Asia is raising, which should be paid more attention. Base on the WHO data, the total per capita alcohol consumption in India increased from 2.4L in 2005 to 5.7L in 2016, while the per capita alcohol consumption in China increased from 4.1L in 2005 to 7.2L in 2016 [1]. Although the alcohol consumption in India was lower than that in China, the prevalence in India was significantly higher than that in China, which was related to socio-economic factors.

Smoking is an independent risk factor for liver fibrosis that could accelerate the natural course of ARLD [181]. Whether a dose-related correlation was established between tobacco and the disease and whether the progression of ARLD was related to smoking years needs to be confirmed further by a large number of studies. Based on the current results, > 50% of ARLD had former or current smoking. The harmful effect of smoking on liver-related diseases should be under intensive focus. Hepatitis virus infection and alcohol are the main pathogenic factors of chronic liver injury; their co-existence could aggravate liver damage through virus replication and immune suppression [406, 413]. Therefore, treating the co-existent viral hepatitis with antiviral drugs is imperative.

Some studies proposed that moderate drinking was beneficial to health [414–416], but the defined alcohol intake and types of alcohol have not reached a consensus. American Association for the Study of Liver Diseases has set a safe threshold of alcohol consumption for men (no more than 2standard drinks per 24 h) and women (no more than 1standard drinks per 24 h) [417]. Meanwhile, the research results have shown that the longer the drinking years, the more patients suffer from ARLD. Early abstinence from alcohol is crucial to reducing the risk of ARLD.

While analyzing the influence of race on ARLD, we found that the proportion varies greatly among different subgroups, especially Caucasians and Asians. Notably, the percentage of the minority group with ARLD was much higher than the Han group. This phenomenon could be attributed to the difference in the constitution and the cultural customs; whether we could improve the early detection rate by setting different safety thresholds for different ethnicities needs to be investigated further.

The advantages of this study were to provide as complete as possible the epidemiological characteristics of ARLD. Presently, the epidemiological investigations of ARLD are mainly regional studies. This study provided the necessary data for countries to study ARLD and provided the trend of the disease to help the government in formulating alcohol management policies and conducting public education. Nevertheless, the present study had some limitations: the high heterogeneity between each study, which is common in such meta-analysis; only 1 or 2 datasets were included in subgroups resulting in the limitations of the results.

The eight studies to calculate the incidence are mainly concentrated in Europe, where the alcoholic population is concentrated, so the results are relatively high. Large cohort study, which cost a lot of manpower and material resources, is very important to explore the global incidence. Based on the premise of large population base and long follow-up time, the results of this review can reflect the global incidence to a certain extent. The phenomenon—mortality deduced from the 22 datasets was used to analyze the cause of death, which was much higher than that of 114 studies used for the analysis of mortality. This phenomenon could be attributed to the following reasons: the populations in the articles providing the cause of death were serious; the sample size was small; the study population was concentrated in European countries. Therefore, 23.9% was similar to the level of global mortality.

Taken together, these results showed that ARLD is one of the most common chronic liver diseases in the world, with a prevalence of 4.8%. With an improved economy, the per capita alcohol consumption is increasing rapidly, and the increasing risk of ARLD in developing countries is gaining much attraction. In addition to strengthening the management of abstinence from alcohol, we should investigate the metabolism, histology, and clinical characteristics of ARLD. Also, we should get rid of the dependence on asking the patients about their drinking history for diagnosis. Thus, non-invasive examinations and specific biomarkers are essential for early recognition. In addition to banning alcohol intake, developing safe and effective intervention measures, such as using the gut-liver axis with probiotics and prebiotics to improve the gut microbiota. Nonetheless, there are still many issues with respect to the prevention, development, and prognosis of ARLD that need to be investigated to improve the epidemiology of ARLD.

## Conclusions

Overall, the global prevalence of ARLD is 4.8%, and the prevalence varies greatly among different regions, which may be influenced by various factors such as gender, race/ethnicity, drinking years, comorbidities and so on. With the improvement of economic level, the prevalence of ARLD is on the rise. By increasing alcoholic taxes and controlling the quantity and timing of alcohol sales, the harm caused by alcohol can be reduced to some extent. Large—scale cross-sectional and cohort studies are helpful to understand the epidemiological characteristics of ARLD.

## Supplementary Information

**Additional file 1:** **Supplementary methods. Supplementary Table 1.** Search strategy. **Supplementary Table 2.** Characteristics of included studies for analysis of overall ARLD prevalence. **Supplementary Table 3.** Summary of articles used to analyze the characteristics of ARLD. **Supplementary Table 4.** Characteristics of included studies for analysis of overall ARLD incidence. **Supplementary Table 5.** Characteristics of included studies for analysis of overall ARLD Mortality. **Supplementary Figure 1.** The forest plots of global prevalence. **Supplementary Figure 2.** The forest plots of China prevalence. **Supplementary Figure 3.** The forest plots of men prevalence. **Supplementary Figure 4.** The forest plots of female prevalence. **Supplementary Figure 5.** Funnel plot of studies included for analysis of overall ARLD prevalence.

## Data Availability

The data that support the findings of this study are available from the corresponding author.

## Abbreviations

ARLD: Alcohol related liver disease
CNKI: China National Knowledge Infrastructure
NIAAA: National Institute on Alcohol Abuse and Alcoholism
SBP: Spontaneous peritonitis
