# Correction: Global prevalence, incidence, and outcomes of alcohol related liver diseases: a systematic review and meta-analysis

**DOI:** 10.1186/s12889-023-15986-0

**Published:** 2023-07-18

**Authors:** Xuanxuan Niu, Lin Zhu, Yifan Xu, Menghan Zhang, Yanxu Hao, Lei Ma, Yan Li, Huichun Xing

**Affiliations:** 1 Center of Liver Diseases Division 3, Beijing Ditan Hospital, Capital Medical University, 8 Jingshundong Street, Chaoyang District, 100015 Beijing, China; 2 Peking University Ditan Teaching Hospital, Beijing, 100015 China

**Correction: BMC Public Health 23, 859 (2023)**

**https://doi.org/10.1186/s12889-023-15749-x**

The original publication of this article [1] contained several transcription errors as a result of importing data from the statistical software. The correct data calculation was used for the results & conclusions of the paper. The incorrect & correct information is listed in this correction article, the original article has been updated.

**Table:** 

Section	Incorrect	Correct
Results	Finally, 372 articles were included: **352** studies for prevalence analysis, 7 for incidence analysis, and 114 for outcome analysis (some studies provided data on prevalence, incidence and/or outcomes at the same time; hence, the total number was different from the sum of subgroups) (Fig. 1)	Finally, 372 articles were included: **353** studies for prevalence analysis, 7 for incidence analysis, and 114 for outcome analysis (some studies provided data on prevalence, incidence and/or outcomes at the same time; hence, the total number was different from the sum of subgroups) (Fig. 1)
	The prevalence rates were relatively low in Sichuan (1.8%, 95% CI: 1.1–2.6), Beijing (1.9%, 95% CI: 0.1–**2.6)**, Guangdong (1.9%, 95% CI: 0.7–3.6), Jiangsu (2.2%, 95% CI: 1.9–2.5), and Shanghai (2.9%, 95% CI: 2.5–3.3), all which were relatively economically developed regions (Table 1)	The prevalence rates were relatively low in Sichuan (1.8%, 95% CI: 1.1–2.6), Beijing (1.9%, 95% CI: 0.1–**5.5**), Guangdong (1.9%, 95% CI: 0.7–3.6), Jiangsu (2.2%, 95% CI: 1.9–2.5), and Shanghai (2.9%, 95% CI: 2.5–3.3), all which were relatively economically developed regions (Table 1)
	Herein, **62** studies [14–16, 18–20, 22–25, 29–31, 33– 40, 44–46, 50, 53, 55, 56, 58–60, 63–67, 71, 77, 78, 80, 81, 84–86, 92, 93, 96, 97, 99–101, 104–107, 111] were included to analyze the influence of sex on the prevalence of ARLD. The prevalence of male was 2.9% (95% CI: 2.4– 3.5), which was much higher than that of women (0.5%, 95% CI: 0.4–0.7) (Table 1)	Herein, **58** studies [14–16, 18–20, 22–25, 29–31, 33– 40, 44–46, 50, 53, 55, 56, 58–60, 63–67, 71, 77, 78, 80, 81, 84–86, 92, 93, 96, 97, 99–101, 104–107, 111] were included to analyze the influence of sex on the prevalence of ARLD. The prevalence of male was 2.9% (95% CI: 2.4– 3.5), which was much higher than that of women (0.5%, 95% CI: 0.4–0.7) (Table 1)
	Three of the **69** articles [210, 235, 236] described that the liver damage caused by concurrent hepatitis virus infection and alcohol was severe	Three of the **67** articles [210, 235, 236] described that the liver damage caused by concurrent hepatitis virus infection and alcohol was severe
	A total of **121** datasets [25, 26, 30, 43, 47, 113, 115, 116, 122, 125, 126, 128, 129, 131, 132, 136, 137, 142, 153, 156, 157, 161, 162, 168, 171–173, 176, 178, 186, 190, 193–197, 204, 207, 208, 213, 215, 221, 225–230, 241–243, 245, 250–252, 255, 262, 267, 272, 275, 276, 294, 297, 299, 306, 311, 318, 321–372] were used to analyze the prevalence of ascites, gastrointestinal bleeding, hepatic encephalopathy, spontaneous peritonitis (SBP), hepatorenal syndrome, and bacterial infection in ARLD	A total of **119** datasets [25, 26, 30, 43, 47, 113, 115, 116, 122, 125, 126, 128, 129, 131, 132, 136, 137, 142, 153, 156, 157, 161, 162, 168, 171–173, 176, 178, 186, 190, 193–197, 204, 207, 208, 213, 215, 221, 225–230, 241–243, 245, 250–252, 255, 262, 267, 272, 275, 276, 294, 297, 299, 306, 311, 318, 321–372] were used to analyze the prevalence of ascites, gastrointestinal bleeding, hepatic encephalopathy, spontaneous peritonitis (SBP), hepatorenal syndrome, and bacterial infection in ARLD
Table 1	5.0% (4.3–5.8)	4.8%(4.1–5.6)
Table 2	**69**	**67**
Figure 2 caption	The global prevalence of ARLD. The prevalence in 14 countries was indicated by depth of red. The **15** countries included Portugal, Canada, Iceland, France, China, USA, Denmark, South Korea, Uganda, India, UK, Sweden, Japan, and Italy	The global prevalence of ARLD. The prevalence in 14 countries was indicated by depth of red. The **14** countries included Portugal, Canada, Iceland, France, China, USA, Denmark, South Korea, Uganda, India, UK, Sweden, Japan, and Italy
Figure 3 caption	The prevalence of ARLD in China by provinces. The prevalence in 21 provinces of China was indicated by depth of red. The prevalence in China was obtained from the analysis of **19** cities or provinces including Sichuan, Beijing, Guangdong, Jiangsu, Shanghai, Gansu, Shaanxi, Guizhou, Zhejiang, Henan, Hunan, Jilin, Heilongjiang, Taiwan, Tibet, Liaoning, Yunnan, Anhui, Shandong, Hebei, and Xinjiang provinces	The prevalence of ARLD in China by provinces. The prevalence in 21 provinces of China was indicated by depth of red. The prevalence in China was obtained from the analysis of **21** cities or provinces including Sichuan, Beijing, Guangdong, Jiangsu, Shanghai, Gansu, Shaanxi, Guizhou, Zhejiang, Henan, Hunan, Jilin, Heilongjiang, Taiwan, Tibet, Liaoning, Yunnan, Anhui, Shandong, Hebei, and Xinjiang provinces
Discussion	Taken together, these results showed that ARLD is one of the most common chronic liver diseases in the world, with a prevalence of **5.8%**	Taken together, these results showed that ARLD is one of the most common chronic liver diseases in the world, with a prevalence of **4.8%**
