# Supplementary material for: Global prevalence, incidence, and outcomes of alcohol related liver diseases: a systematic review and meta-analysis
Source: BMC Public Health. 2023 May 11;23:859. doi: 10.1186/s12889-023-15749-x (PMC10173666; doi:10.1186/s12889-023-15749-x)

**Supplementary methods**

**Supplementary Table. 1 Search strategy**

Pubmed, from inception to May 31, 2022(13310 articles)

| **No.** | **Search terms** | **Results** |
| --- | --- | --- |
| #1 | **("Liver Diseases, Alcoholic"[Mesh]) OR (((Alcoholic Liver Diseases[Title/Abstract]) OR (Alcoholic Liver Disease[Title/Abstract])) OR (Liver Disease, Alcoholic[Title/Abstract]))** | 18489 |
| #2 | **("Fatty Liver, Alcoholic"[Mesh]) OR ((Alcoholic Fatty Liver[Title/Abstract]) OR (Alcoholic Steatohepatitis[Title/Abstract]))** | 18229 |
| #3 | **("Hepatitis, Alcoholic"[Mesh]) OR (((((Alcoholic Hepatitis[Title/Abstract]) OR (Chronic Alcoholic Hepatitis[Title/Abstract])) OR (Alcoholic Hepatitis, Chronic[Title/Abstract])) OR (Chronic Alcoholic Hepatitides[Title/Abstract])) OR (Hepatitis, Alcoholic, Chronic[Title/Abstract]))** | 4040 |
| #4 | **("Liver Cirrhosis, Alcoholic"[Mesh]) OR ((((Alcoholic Liver Cirrhosis[Title/Abstract]) OR (Hepatic Cirrhosis, Alcoholic[Title/Abstract])) OR (Alcoholic Hepatic Cirrhosis[Title/Abstract])) OR (Alcoholic Cirrhosis[Title/Abstract]))** | 9495 |
| #5 | **((((((((((("Non-alcoholic Fatty Liver Disease"[Mesh]) OR (Non alcoholic Fatty Liver Disease)) OR (NAFLD)) OR (Nonalcoholic Fatty Liver Disease)) OR (Fatty Liver, Nonalcoholic)) OR (Fatty Livers, Nonalcoholic)) OR (Liver, Nonalcoholic Fatty)) OR (Livers, Nonalcoholic Fatty)) OR (Nonalcoholic Fatty Liver)) OR (Nonalcoholic Fatty Livers)) OR (Nonalcoholic Steatohepatitis)) OR (Steatohepatitis, Nonalcoholic)** | 35643 |
| #6 | **((((((randomized controlled trial[Publication Type]) OR (controlled clinical trial[Publication Type])) OR (randomized[Title/Abstract])) OR (clinical trials as topic[MeSH])) OR (clinical trial[Title/Abstract])) OR (clinical trials[Title/Abstract])) OR (clinical trial[Publication Type])** | 1667776 |
| #7 | **(animals[Mesh]) NOT (humans[Mesh])** | 5010558 |
| #8 | **((mice [Title/Abstract]) OR (rat[Title/Abstract])) OR (murine[Title/Abstract])** | 1988844 |
| #9 | **((review[Publication Type]) OR (meta analysis[Publication Type])) OR (systematic review[Publication Type])** | 3130434 |
| #10 | #1 OR #2 | 34350 |
| #11 | #10 OR #3 | 35160 |
| #12 | #11 OR #4 | 36848 |
| #13 | #12 NOT #5 | 19391 |
| #14 | #13 NOT #6 | 18399 |
| #15 | #14 NOT #7 | 16409 |
| #16 | #15 NOT #8 | 15709 |
| #17 | #16 NOT #9 | 13310 |

Embase, from inception to May 31, 2022(42582 articles)

| **No.** | **Search terms** | **Results** |
| --- | --- | --- |
| **#1** | 'liver diseases, alcoholic' OR 'alcoholic liver diseases':ab,ti OR 'alcoholic liver disease':ab,ti OR 'liver disease, alcoholic':ab,ti | 11314 |
| **#2** | 'fatty liver, alcoholic' OR 'alcoholic fatty liver':ab,ti OR 'alcoholic steatohepatitis':ab,ti | 29433 |
| **#3** | 'hepatitis, alcoholic' OR 'alcoholic hepatitis':ab,ti OR 'chronic alcoholic hepatitis':ab,ti OR 'alcoholic hepatitis, chronic':ab,ti OR 'chronic alcoholic hepatitides':ab,ti OR 'hepatitis, alcoholic, chronic':ab,ti | 5445 |
| **#4** | 'liver cirrhosis, alcoholic':ab,ti OR 'alcoholic liver cirrhosis':ab,ti OR 'hepatic cirrhosis, alcoholic':ab,ti OR 'alcoholic hepatic cirrhosis':ab,ti OR 'alcoholic cirrhosis':ab,ti | 7369 |
| **#5** | #1 OR #2 | 15263 |
| **#6** | #5 OR #3 | 21357 |
| **#7** | #6 OR #4 | 48885 |
| **#8** | (#4 OR #6) AND [embase]/lim AND ([chinese]/lim OR [english]/lim) | 42582 |

Cochrane library, from inception to May 31, 2022 (474 articles)

| **No.** | Search terms | Results |
| --- | --- | --- |
| **#1** | MeSH descriptor: [Liver Diseases, Alcoholic] explode all trees | 503 |
| **#2** | MeSH descriptor: [Fatty Liver, Alcoholic] explode all trees | 22 |
| **#3** | MeSH descriptor: [Hepatitis, Alcoholic] explode all trees | 155 |
| **#4** | MeSH descriptor: [Liver Cirrhosis, Alcoholic] explode all trees | 234 |
| **#5** | (review):pt OR (meta analysis): OR (systematic review):pt | 19690 |
| **#6** | (mice):ti,ab,kw OR (rat):ti,ab,kw | 9115 |
| **#7** | #1 or #2 | 503 |
| **#8** | #7 or #3 | 503 |
| **#9** | #8 or #4 | 503 |
| **#10** | #9 not #5 | 493 |
| **#11** | #10 not #6 | 484 |

**Supplementary Table. 2** Characteristics of included studies for analysis of overall ARLD prevalence

| Author | Publication year | Study period | Study country/city | Mean/median age | Population (N) | ARLD (n) | Male  (n) | Prevalence (%) | Total score (0-9) |
| --- | --- | --- | --- | --- | --- | --- | --- | --- | --- |
| Saunders JB | 1981 | 1959-1976 | UK | - | 275000 | 242 | - | 0.09 | 7 |
| Huang C | 2018 | 2012 | China(Anhui) | 59.80 | 3393 | 101 | 40 | 2.98 | 8 |
| Klatsky AL | 1992 | 1978-1985 | USA | 48.50 | 128934 | 68 | 42 | 0.05 | 7 |
| Singal AK | 2021 | 2006-2016 | USA | - | 2264364 | 593508 | 426986 | 26.21 | 8 |
| Dang K | 2020 | 2001-2016 | USA | 53.94 | 44631 | 3467 | - | 7.77 | 9 |
| Hsieh PH | 2017 | 2001-2008 | China(Taiwan) | 47.00 | 46565 | 2249 | 2091 | 4.83 | 8 |
| Adejumo AC | 2018 | 2014-2014 | USA | - | 319514 | 64207 | 45487 | 20.10 | 9 |
| Hamaguchi M | 2020 | 2014-2017 | Japan | 50.00 | 20029 | 223 | - | 1.11 | 9 |
| Bao, X. Y. | 2015 | 2006-2010 | China(Beijing) | - | 2290144 | 3523 | 3457 | 0.15 | 8 |
| Duchmann JC | 1997 | 1993-1994 | France | 52.10 | 2138 | 40 | 25 | 1.87 | 8 |
| Klatsky AL | 2006 | 1978-2001 | USA | - | 125580 | 199 | 129 | 0.16 | 9 |
| Ozaki K | 2013 | 2002-2009 | Japan | 62.20 | 879 | 116 | 99 | 13.20 | 8 |
| Tao N | 2003 | 1999-1999 | USA | - | 609745 | 2084 | 1554 | 0.34 | 9 |
| Huang A | 2017 | 2002-2013 | China(Beijing) | - | 188902 | 7422 | 7281 | 3.93 | 8 |
| Bellentani S | 1997 | - | Italy | - | 6917 | 74 | 67 | 1.07 | 7 |
| Piette JD | 1998 | - | USA | 48.00 | 46680 | 5627 | - | 12.05 | 7 |
| Singal AK | 2012 | 1998-2007 | USA | - | 76957719 | 111726 | 79566 | 0.15 | 8 |
| Hislop WS | 2004 | 2000-2001 | UK | - | 1323 | 191 | 120 | 14.44 | 6 |
| Lee JY | 2019 | 2008-2012 | South Korea | - | 48900000 | 39800 | 35552 | 0.08 | 9 |
| Hauksson K | 2020 | 2001-2015 | Iceland | 57.00 | 16000 | 159 | 60 | 0.99 | 9 |
| Singal AK | 2020 | 2006-2014 | USA | - | 1928764 | 447090 | - | 23.18 | 7 |
| Goldacre MJ | 2008 | 1963-1999 | UK | - | 599308 | 4080 | 2600 | 0.68 | 7 |
| Cortez-Pinto H | 2004 | 1995-2001 | Portugal | 57.00 | 12371 | 7751 | 6120 | 62.65 | 7 |
| Nilsson E | 2019 | 2001-2017 | Sweden | 60.34 | 1317 | 645 | - | 48.97 | 8 |
| Trimble G | 2013 | 1988-1994 | USA | 41.54 | 8306 | 148 | 114 | 1.78 | 7 |
| Capocaccia L | 1994 | 1988-1989 | Italy | - | 1402 | 504 | 442 | 35.95 | 8 |
| Horie Y | 2013 | 2007-2008 | Japan | 57.20 | 16224 | 2070 | 1843 | 12.76 | 8 |
| Cuthbert JA | 2014 | 2002-2005 | USA | 45.00 | 1761 | 148 | 118 | 8.40 | 8 |
| Becker U | 1996 | 1976-1978 | Denmark | - | 13285 | 385 | 280 | 2.90 | 8 |
| Wong T | 2019 | 2001-2006 | USA | 40.20 | 34423 | 1480 | 897 | 4.30 | 8 |
| Park SH | 2011 | 2009 | South Korea | - | 7517 | 109 | 80 | 1.45 | 9 |
| Haixia Wang | 2014 | 2011 | China(Shandong) | - | 7295 | 624 | 572 | 8.55 | 7 |
| Iritani S | 2022 | 2019-2020 | Japan | 64.00 | 666 | 77 | - | 11.56 | 8 |
| Tapper EB | 2016 | 2011 | USA | 60.90 | 130455 | 45268 | - | 34.70 | 8 |
| Nishikawa H | 2021 | 2014-2019 | Japan | 68.00 | 1624 | 156 | - | 9.61 | 7 |
| Dam MK | 2013 | - | Denmark | 50.46 | 18479 | 225 | 160 | 1.22 | 9 |
| Garg R | 2013 | 2010 | India | - | 2310 | 160 | - | 6.93 | 8 |
| Jepsen P | 2016 | 1994-2015 | Denmark | 56.40 | 5500000 | 10650 | 7455 | 0.19 | 9 |
| Liangpunsakul S | 2011 | 2007 | USA | 53.20 | 8043415 | 56809 | 41471 | 0.71 | 9 |
| Nguyen TA | 2016 | 2000-2011 | USA | 48.00 | 8130198 | 6113 | 4208 | 0.08 | 8 |
| Jinjuvadia R | 2015 | 2002-2010 | USA | 53.20 | 39008298 | 326403 | 237621 | 0.84 | 9 |
| Green MA | 2017 | 2002-2013 | UK | - | 303716 | 16142 | - | 5.31 | 8 |
| Roberts SE | 2005 | 1968-1999 | UK | 53.30 | 8192 | 2802 | 1717 | 34.20 | 8 |
| Ray G | 2014 | 2003-2011 | India | - | 919 | 262 | - | 28.51 | 8 |
| Wang H | 2022 | 2017-2020 | China(Beijing) | 49.45 | 74988 | 974 | 952 | 1.30 | 9 |
| Gonzalez HC | 2022 | 2016-2020 | USA | 46.18 | 146498 | 337 | 156 | 0.23 | 8 |
| Jin, L. | 2008 | 2006 | China(Zhejiang) | 48.00 | 2536 | 55 | - | 2.17 | 8 |
| Liu, X. S. | 2014 | 2010-2012 | China(Guangdong) | 45.00 | 6010 | 366 | - | 6.09 | 7 |
| Zhu, B. | 2015 | 2003-2012 | China(Beijing) | 48.11 | 106308 | 4132 | - | 3.89 | 9 |
| Deng, H. X. | 2010 | 2008-2009 | China(Gansu) | 43.60 | 3481 | 69 | - | 1.98 | 8 |
| Yan, H. | 2015 | - | China(Shaanxi/Gansu/Xinjiang) | 38.10 | 2300 | 201 | 174 | 8.74 | 7 |
| Long, X. | 2012 | 2011-2012 | China(Guizhou) | 45.90 | 16062 | 590 | - | 3.67 | 9 |
| Shen, F. Q. | 2009 | 2008 | China(Zhejiang) | - | 2212 | 36 | 29 | 1.63 | 6 |
| Huang, W. Y. | 2011 | 2007 | China(Anhui) | - | 2880 | 480 | - | 16.67 | 6 |
| Yang, X. X. | 2009 | 2007-2008 | China(Sichuan) | 47.90 | 12152 | 299 | 299 | 2.46 | 8 |
| Qin, L. | 2012 | 2011 | China(Shanghai) | 55.60 | 3017 | 142 | - | 4.71 | 8 |
| Wu, J. L. | 2003 | 1995-2001 | China(Heilongjiang) | - | 1203 | 58 | - | 4.82 | 7 |
| Sun, J. | 2008 | 2007 | China(Jilin) | 43.89 | 3815 | 152 | - | 3.98 | 9 |
| Ma, J. X. | 2007 | 2005 | China(Guangdong) | 40.52 | 2043 | 38 | 30 | 1.86 | 9 |
| Cui, L. | 2010 | 2009-2010 | China(Guizhou) | 48.00 | 4167 | 186 | 186 | 4.46 | 8 |
| Wang, S. Q. | 2003 | 2000-2003 | China(Henan) | - | 4358 | 211 | - | 4.84 | 8 |
| Huang, S. L. | 2005 | - | China(Hunan) | 42.10 | 18618 | 811 | 781 | 4.36 | 9 |
| Li, T. M. | 2001 | 1999 | China(Hebei) | - | 866 | 85 | - | 9.82 | 7 |
| Chen, S. R. | 2008 | 2006-2007 | China(Guangdong) | 44.60 | 3483 | 71 | 71 | 2.04 | 8 |
| Zhao, Y. X. | 2017 | 2011-2014 | China(Gansu) | 46.99 | 46861 | 1558 | 1546 | 3.32 | 8 |
| Yuan, Q. Y. | 2011 | 2008-2010 | China(Liaoning) | 36.50 | 7420 | 368 | - | 4.96 | 9 |
| Yan, X. H. | 2010 | - | China(Shandong) | 42.30 | 1200 | 68 | 65 | 5.67 | 7 |
| Zhou. Y. F. | 2001 | 2000 | China(Zhejiang) | - | 4500 | 521 | - | 11.58 | 9 |
| Hou, S. K. | 2011 | - | China(Sichuan) | - | 1360 | 8 | 8 | 0.59 | 7 |
| Sun, M. | 2005 | 2004 | China(Jiangsu) | - | 4542 | 137 | - | 3.02 | 8 |
| He, W. S. | 2006 | 2001-2005 | China(Guangdong) | 58.10 | 14069 | 664 | - | 4.72 | 8 |
| Yan, H. | 2007 | 2005 | China(Shaanxi and Gansu) | 38.26 | 1500 | 58 | 51 | 3.87 | 7 |
| Fan, J. G. | 2005 | 2002-2003 | China(Shanghai) | 52.40 | 3157 | 50 | - | 1.58 | 8 |
| Zhou, J. H. | 2008 | 2007 | China(Sichuan) | - | 1000 | 16 | - | 1.60 | 7 |
| Liu, Y. P. | 2008 | 2005-2007 | China(Sichuan) | 42.97 | 8756 | 271 | 268 | 3.10 | 8 |
| Yang, Z. L. | 2008 | 2005-2006 | China(Sichuan) | 42.42 | 4504 | 61 | - | 1.35 | 8 |
| Xing, Y. | 2011 | 2008 | China(Xinjiang) | - | 2567 | 274 | - | 10.67 | 9 |
| Lu, X. L. | 2003 | 2000 | China(Shaanxi) | 36.00 | 3613 | 83 | - | 2.30 | 8 |
| Baima, K. Z. | 2016 | 2009-2010 | China(Tibet) | 43.60 | 2178 | 106 | 86 | 4.87 | 8 |
| Ye, G. L. | 2005 | 1999 | China(Zhejiang) | 38.90 | 4518 | 44 | - | 0.97 | 7 |
| Qiao, L. N. | 2015 | 2011-2013 | China(Shaanxi) | 49.27 | 6236 | 173 | - | 2.77 | 8 |
| Li, Q. N. | 2017 | 2016 | China(Shaanxi) | - | 6732 | 397 | 381 | 5.90 | 9 |
| Yao, J. H. | 2011 | 2010-2011 | China(Yunnan) | 47.89 | 1770 | 84 | - | 4.75 | 8 |
| Li, Y. M. | 2003 | 1999-2000 | China(Zhejiang) | 38.80 | 18237 | 1584 | - | 8.69 | 8 |
| Stroffolini T | 2019 | 2001-2014 | Italy | 55.60 | 11676 | 2628 | - | 22.51 | 8 |
| Mallet V | 2021 | 2020 | France | 70.00 | 259110 | 3623 | - | 1.40 | 7 |
| Sheng, D. P. | 2003 | 2001-2002 | China(zhejiang) | 32.00 | 3911 | 53 | - | 1.36 | 8 |
| Yan, L. J. | 2003 | 2001 | China(Hunan) | 52.00 | 1078 | 25 | - | 2.32 | 6 |
| Wu, Y. P. | 2012 | 2008-2009 | China(Yunnan) | 49.00 | 500 | 75 | - | 15.00 | 7 |
| Enomoto H | 2020 | 2008-2016 | Japan | 66.40 | 48621 | 9676 | - | 19.90 |  |
| Zhang, Z. D. | 2009 | 2003-2007 | China(Zhejiang) | 30.50 | 2126 | 40 | - | 1.88 | 8 |
| Cao, Z. Y. | 2001 | 2000 | China(Jilin) | - | 234 | 11 | - | 4.70 | 8 |
| Kuang, Z. P. | 2011 | 2006-2010 | China(Guangdong) | 68.90 | 2230 | 35 | - | 1.57 | 7 |
| Wang, J. | 2008 | 2006 | China(Henan) | - | 1290 | 30 | - | 2.33 | 9 |
| Pen, M. X. | 2011 | 2009-2010 | China(Zhejiang) | 44.75 | 3246 | 416 | - | 12.82 | 8 |
| Shi, X. D. | 2011 | 2007 | China(Jilin) | - | 3815 | 143 | 138 | 3.75 | 8 |
| Chen, S. L. | 2010 | 2007 | China(Liaoning) | 40.52 | 18020 | 450 | 400 | 2.50 | 7 |
| Slaunwhite AK | 2015 | 2001-2011 | Canada | 45.90 | 690401 | 7112 | 4846 | 1.03 | 7 |
| O'Hara G | 2020 | 2011 | Uganda | 30.00 | 8099 | 888 | - | 10.96 | 6 |

**Supplementary Table. 3** Summary of articles used to analyze the characteristics of ARLD.

| Author | Publication year | Study period | Study country/city | Mean/median age | ARLD (n) | Male  (n) | Female  (n) | Male (%) | Total score  (0-9) |
| --- | --- | --- | --- | --- | --- | --- | --- | --- | --- |
| Nalpas B | 1997 | - | France | 45.39 | 138 | 94 | 44 | 68.12 | 6 |
| Sujan R | 2018 | - | USA/Europe/India | 47.00 | 773 | 633 | 140 | 81.89 | 8 |
| Björnsson ES | 2020 | 2001-2016 | Iceland | 56.00 | 158 | 114 | 44 | 72.15 | 8 |
| Santos SGRD | 2018 | 2009-2014 | Brazil | 55.60 | 152 | 144 | 8 | 94.74 | 7 |
| Cichoz-Lach H | 2007 | - | Poland | 49.99 | 57 | 39 | 18 | 68.42 | 6 |
| Klatsky AL | 1992 | 1978-1985 | USA | 48.50 | 68 | 42 | 26 | 61.76 | 7 |
| Singal AK | 2021 | 2006-2016 | USA | - | 593508 | 426986 | 166522 | 71.94 | 8 |
| Otete HE | 2016 | 1997-2012 | UK | 55.60 | 2479 | 1660 | 819 | 66.96 | 7 |
| Spicak J | 2012 | 2009-2012 | Czech Republic | 47.50 | 80 | 64 | 16 | 80.00 | 8 |
| Dang K | 2020 | 2001-2016 | USA | 53.94 | 3467 | - | - | - | 9 |
| Pradhan B | 2015 | 2009 | Nepal | 53.50 | 447 | 292 | 155 | 65.32 | 8 |
| Nakamura S | 1979 | 1972-1977 | Japan | - | 130 | 123 | 7 | 94.62 | 7 |
| Hourigan KJ | 2001 | 1978-1997 | Australia | - | 297 | 245 | 52 | 82.49 | 6 |
| Alves PS | 1982 | 1966-1978 | Portugal | - | 463 | 339 | 124 | 73.22 | 7 |
| Ravi S | 2017 | 2004-2012 | UK | 47.99 | 105 | 69 | 36 | 65.71 | 7 |
| Mancebo A | 2013 | 1992-2010 | Spain | 54.10 | 264 | 225 | 39 | 85.23 | 8 |
| Poynard T | 1986 | 1982-1985 | France | - | 681 | 369 | 312 | 54.19 | 8 |
| Faizallah R | 1982 | - | - | 50.50 | 170 | 104 | 66 | 61.18 | 7 |
| Lieber CS | 2006 | 1994-1998 | USA | 49.65 | 640 | 625 | 15 | 97.66 | 6 |
| Goyal SK | 2014 | 2011-2012 | India | 44.81 | 104 | - | - | - | 7 |
| Zeng D | 2021 | 2016-2020 | China(Guangdong) | 54.57 | 292 | 283 | 9 | 96.92 | 8 |
| Lee HC | 2001 | - | South Korea | 52.20 | 56 | - | - | - | 7 |
| Hsieh PH | 2017 | 2001-2008 | China(Taiwan) | 47.00 | 2249 | 2091 | 158 | 92.97 | 8 |
| Gluud C | 1983 | - | Denmark | 51.76 | 207 | 179 | 28 | 86.47 | 6 |
| Silva JM | 2021 | 2010-2017 | Portugal | - | 40595 | - | - | - | 9 |
| Adejumo AC | 2018 | 2014 | USA | - | 64207 | 45487 | 18720 | 70.84 | 9 |
| Ratib S | 2015 | 1998-2009 | UK | - | 2756 | 1834 | 922 | 66.55 | 9 |
| Hamaguchi M | 2020 | 2014-2017 | Japan | 50.00 | 223 | - | - | - | 9 |
| Krasner N | 1977 | 1967-1975 | UK | 51.75 | 293 | 215 | 78 | 73.38 | 7 |
| Xie YD | 2013 | 2000-2011 | China(Beijing) | 50.48 | 205 | 204 | 1 | 99.51 | 8 |
| Barritt AS 4th | 2019 | 2010-2012 | USA | 57.00 | 112495 | 80434 | 32061 | 71.50 | 7 |
| Deleuran T | 2012 | 1997-2010 | Denmark | 52.30 | 194 | 99 | 95 | 51.03 | 8 |
| Liangpunsakul S | 2011 | 2007 | USA | 53.20 | 56809 | 41471 | 15338 | 73.00 | 9 |
| Potter JF | 1987 | 1977-1985 | UK | - | 208 | 153 | 55 | 73.56 | 8 |
| Pinon-Gutierrez R | 2017 | 2002-2014 | USA | 48.60 | 201 | 123 | 78 | 61.19 | 7 |
| Fong TL | 1994 | 1991-1992 | USA | 42.00 | 137 | 108 | 29 | 78.83 | 7 |
| Kwon SY | 2000 | 1997-1998 | South Korea | 51.60 | 162 | 160 | 2 | 98.77 | 8 |
| Ray S | 2014 | 2011-2012 | India | - | 200 | - | - | - | 8 |
| Klatsky AL | 2006 | 1978-2001 | USA | - | 199 | 129 | 70 | 64.82 | 9 |
| Toshikuni N | 2009 | 1997-2007 | Japan | 59.00 | 75 | 67 | 8 | 89.33 | 7 |
| Jinjuvadia R | 2018 | 2011 | USA | 53.30 | 74972 | 53380 | 21592 | 71.20 | 7 |
| Ozaki K | 2013 | 2002-2009 | Japan | 62.20 | 116 | 99 | 17 | 85.34 | 8 |
| Tanaka T | 2000 | - | Japan | - | 143 | 132 | 11 | 92.31 | 7 |
| Tao N | 2003 | 1999 | USA | - | 2084 | 1554 | 530 | 74.57 | 9 |
| Vaz K | 2021 | 2010-2019 | Australia | 51.00 | 126 | 72 | 54 | 57.14 | 9 |
| Fan X | 2021 | - | USA | 47.93 | 254 | 162 | 92 | 63.78 | 6 |
| Wiegand J | 2012 | 2002-2007 | France and Germany | - | 328 | 249 | 79 | 75.91 | 7 |
| Huang A | 2017 | 2002-2013 | China(Beijing) | - | 7422 | 7281 | 141 | 98.10 | 8 |
| Serra MA | 2003 | 1973-1997 | Spain | 51.00 | 213 | 183 | 30 | 85.92 | 9 |
| Ganne-Carrié N | 2018 | 2010-2016 | France and Belgian | 58.40 | 652 | 440 | 212 | 67.48 | 8 |
| Douds AC | 2003 | 1987-2000 | - | - | 381 | 177 | 204 | 46.46 | 7 |
| Levy R | 2015 | 2002-2010 | USA | 50.22 | 791 | 526 | 265 | 66.50 | 7 |
| Gonçalves PL | 2013 | 1993-2011 | Brazil | 53.20 | 847 | 787 | 60 | 92.92 | 9 |
| Naveau S | 1997 | 1982-1995 | France | 50.03 | 1604 | 1197 | 407 | 74.63 | 7 |
| Chang C | 2021 | 2014 | China(Taiwan) | 46.00 | 87 | 73 | 14 | 83.91 | 8 |
| Mathurin P | 2007 | 1985-2003 | France | 45.40 | 193 | 155 | 38 | 80.31 | 8 |
| Piette JD | 1998 | - | USA | 48.00 | 5627 | - | - | - | 7 |
| Sagnelli E | 2018 | - | Italy | 55.55 | 1163 | 811 | 352 | 69.73 | 8 |
| Stickel F | 2020 | - | Europe | 57.67 | 2684 | 2128 | 556 | 79.28 | 7 |
| Rajbhandari R | 2015 | 2011 | USA | - | 244383 | 172290 | 72093 | 70.50 | 8 |
| Thuluvath PJ | 2013 | 1998-2006 | USA | - | 112351 | 75837 | 36514 | 67.50 | 9 |
| Parés A | 1990 | - | Spain | 44.40 | 144 | 101 | 43 | 70.14 | 6 |
| Singal AK | 2012 | 1998-2007 | USA | - | 111726 | 79566 | 32160 | 71.22 | 8 |
| Guilera M | 1998 | - | Spain | 43.00 | 104 | 70 | 34 | 67.31 | 7 |
| Waleed M | 2020 | 2006-2014 | USA | 49.50 | 62136 | 41318 | 20818 | 66.50 | 8 |
| Otete H | 2018 | 1997-2014 | UK | 56.00 | 3706 | 2520 | 1186 | 68.00 | 8 |
| Sy AM | 2017 | - | USA | 53.00 | 165 | 138 | 27 | 83.64 | 7 |
| Mills PR | 1988 | 1982 | UK | - | 248 | - | - | - | 8 |
| Gracey M | 1995 | 1989-1991 | Australia | - | 13126 | 8870 | 4256 | 67.58 | 7 |
| Heslin KC | 2017 | 2012 | USA | 53.70 | 72531 | 52309 | 20222 | 72.12 | 7 |
| Singal AK | 2011 | 1993-2008 | USA | 44.86 | 76 | 42 | 34 | 55.26 | 7 |
| Ladhani S | 2021 | 2012-2016 | USA | 46.06 | 130567 | 899730 | 40594 | 68.91 | 7 |
| Saunders JB | 1983 | - | UK | - | 285 | 189 | 96 | 66.32 | 7 |
| Nahon P | 2016 | 2000-2010 | France | 57.80 | 237 | 190 | 47 | 80.17 | 8 |
| Fleming KM | 2008 | 1987-2002 | UK | - | 1287 | - | - | - | 8 |
| Appel-da-Silva MC | 2016 | 2005-2014 | Brazil | - | 203 | - | - | - | 7 |
| Nilsson E | 2016 | 2001-2010 | Sweden | 59.50 | 759 | 552 | 207 | 72.73 | 8 |
| Hauksson K | 2020 | 2001-2015 | Iceland | 57.00 | 159 | 60 | 99 | 37.74 | 9 |
| Kalaitzakis E | 2011 | 1994-2005 | Sweden | 59.00 | 615 | - | - | - | 8 |
| Lamm K | 2021 | 2010-2018 | USA | 46.48 | 108 | 74 | 34 | 68.52 | 6 |
| Gunnarsdottir SA | 2009 | 1994-2003 | Sweden | 58.50 | 563 | - | - | - | 6 |
| Barrio E | 2004 | 1988-1993 | Spain | 42.00 | 256 | 180 | 76 | 70.31 | 8 |
| Louvet A | 2017 | 2002-2015 | France | 49.70 | 398 | 229 | 169 | 57.54 | 8 |
| Dunn W | 2005 | 1995-2001 | USA | 47.00 | 73 | 23 | 50 | 31.51 | 8 |
| Srikureja W | 2005 | 1997-2002 | USA | 43.00 | 202 | 163 | 39 | 80.69 | 8 |
| Israelsen M | 2020 | 2013-2018 | Denmark | 57.00 | 325 | 246 | 79 | 75.69 | 8 |
| Thompson JA | 2018 | 2006-2006 | USA | 54.00 | 15496 | 10537 | 4959 | 68.00 | 8 |
| Trimble G | 2013 | 1988-1994 | USA | 41.54 | 148 | 114 | 34 | 77.03 | 7 |
| Hagström H | 2021 | 1969-2017 | Sweden | 57.10 | 3453 | 2234 | 1219 | 64.70 | 8 |
| Liang W | 2011 | 1999-2004 | Australia | - | 19204 | - | - | - | 9 |
| Husain A | 2020 | 2017-2019 | India | 40.00 | 130 | 126 | 4 | 96.92 | 7 |
| Kim WR | 2001 | 1995 | USA | 50.59 | 229800 | 166356 | 63444 | 72.39 | 8 |
| Peregud DI | 2021 | 2016-2020 | Russia | 53.80 | 110 | 77 | 33 | 70.00 | 8 |
| Guyot E | 2013 | 1999-2011 | France | 57.81 | 279 | 217 | 62 | 77.78 | 8 |
| Fernandes SR | 2017 | 2005-2015 | USA | 51.00 | 170 | 126 | 44 | 74.12 | 9 |
| Cuthbert JA | 2014 | 2002-2005 | USA | 45.00 | 148 | 118 | 30 | 79.73 | 8 |
| Becker U | 1996 | 1976-1978 | Denmark | - | 385 | 280 | 105 | 72.73 | 8 |
| Semb S | 2016 | 1976-1987 | Denmark | 50.40 | 357 | 244 | 113 | 68.35 | 5 |
| Jeong JY | 2018 | 2009-2016 | South Korea | 53.70 | 91 | - | - | - | 7 |
| Park SH | 2011 | 2009-2009 | South Korea | - | 109 | 80 | 29 | 73.39 | 9 |
| Fujimoto M | 1999 | 1988-1997 | Japan | 53.20 | 105 | 95 | 10 | 90.48 | 5 |
| Mendenhall CL | 1984 | - | - | 50.18 | 363 | - | - | - | 6 |
| Lowenfels AB | 1999 | 1989-1994 | Portugal | 57.45 | 820 | 629 | 191 | 76.71 | 5 |
| Levi AJ | 1978 | 1971-1977 | UK | 53.15 | 202 | 144 | 58 | 71.29 | 7 |
| Tsutsumi M | 1996 | 1985-1995 | Japan | - | 558 | 457 | 101 | 81.90 | 8 |
| Pathak OK | 2009 | 2005-2007 | Nepal | 52.08 | 181 | 146 | 35 | 80.66 | 8 |
| Arun AC | 2020 | 2019-2019 | India | - | 77 | - | - | - | 7 |
| N'Kontchou G | 2006 | 1994-2004 | France | 59.45 | 551 | 339 | 212 | 61.52 | 8 |
| Pang JX | 2015 | 2008-2012 | Canada | 49.00 | 122 | 73 | 49 | 59.84 | 9 |
| Tan JH | 2020 | 2008-2018 | China(Guangdong) | 53.40 | 647 | 633 | 14 | 97.84 | 7 |
| Bruno Raynard | 2002 | - | France | 52.00 | 268 | 208 | 60 | 77.61 | 8 |
| Hagström H | 2021 | 2015-2017 | Sweden | 57.20 | 3410 | 2293 | 1117 | 67.24 | 9 |
| Beaudoin JJ | 2021 | - | USA | 46.30 | 211 | 125 | 86 | 59.24 | 7 |
| Wagnerberger S | 2006 | - | Germany | 48.67 | 118 | 99 | 19 | 83.90 | 8 |
| Nøjgaard C | 2003 | - | Europe | 49.00 | 370 | 277 | 93 | 74.86 | 7 |
| Nault JC | 2013 | 2007-2009 | France | 62.00 | 295 | 253 | 42 | 85.76 | 8 |
| Dam MK | 2013 | - | Denmark | 50.46 | 225 | 160 | 65 | 71.11 | 9 |
| Huang HH | 2008 | 1997-2006 | China(Taiwan) | 45.56 | 1209 | - | - | - | 7 |
| Brunt PW | 1974 | 1960-1967 | UK | 50.12 | 258 | 183 | 75 | 70.93 | 5 |
| Wang X | 2014 | 2009-2011 | India | 45.00 | 722 | 703 | 19 | 97.37 | 8 |
| Huang YS | 2016 | - | China(Guangdong) | 57.20 | 80 | 47 | 33 | 58.75 | 7 |
| Bhattacharyya M | 2016 | 2009-2011 | India | 45.00 | 722 | 703 | 19 | 97.37 | 7 |
| Silva MJ | 2015 | 2003-2012 | Portugal | 52.98 | 52174 | - | - | - | 8 |
| Sofair AN | 2010 | 1999-2001 | USA | - | 82 | 59 | 23 | 71.95 | 6 |
| Makar M | 2021 | - | USA | 59.40 | 29906 | 24782 | 5124 | 82.87 | 8 |
| May FP | 2016 | 2008-2011 | USA | 47.00 | 11304 | 7015 | 4289 | 62.06 | 7 |
| Nasir M | 2020 | 2012-2015 | USA | 50.77 | 132897 | 92139 | 40758 | 69.33 | 9 |
| Said A | 2004 | 1994-2001 | USA | 50.44 | 724 | 509 | 215 | 70.30 | 9 |
| Sersté T | 2018 | 2006-2015 | Belgium | - | 165 | 108 | 57 | 65.45 | 8 |
| Bouttell J | 2016 | 1991-2011 | UK | - | 35208 | 24008 | 11200 | 68.19 | 8 |
| Altamirano J | 2014 | 2000-2008 | Spain | 49.00 | 121 | 78 | 43 | 64.46 | 7 |
| Altamirano J | 2011 | 2006-2008 | Mexico | 43.00 | 175 | 149 | 26 | 85.14 | 8 |
| Al-Azzawi Y | 2020 | - | USA | 47.60 | 196 | 136 | 60 | 69.39 | 5 |
| Alvarez MA | 2011 | 1998-2001 | Spain | 56.00 | 165 | 135 | 30 | 81.82 | 9 |
| Atkinson SR | 2020 | - | Denmark | - | 824 | 520 | 304 | 63.11 | 7 |
| Beck JI | 2017 | 2006-2010 | UK | 48.00 | 82 | 49 | 33 | 59.76 | 8 |
| Božin T | 2021 | 2014-2018 | Croatia | 55.80 | 70 | 52 | 18 | 74.29 | 8 |
| Campollo O | 2001 | 1995-1996 | Mexico | 47.45 | 124 | 104 | 20 | 83.87 | 8 |
| Caregaro L | 1996 | 1991-1994 | Italy | 56.90 | 77 | 61 | 16 | 79.22 | 6 |
| Charni F | 2011 | 1995-2005 | France | 55.00 | 253 | 185 | 68 | 73.12 | 8 |
| Daswani R | 2018 | 2015-2017 | India | 41.00 | 183 | 180 | 3 | 98.36 | 6 |
| Deleuran T | 2016 | 1996-2014 | Denmark | 57.00 | 22734 | 15686 | 7048 | 69.00 | 9 |
| Deltenre P | 2002 | 1999-1993 | France | 49.76 | 89 | 60 | 29 | 67.42 | 6 |
| Fan X | 2020 | 2000-2017 | USA | 54.40 | 1892271 | 1381660 | 510611 | 73.02 | 8 |
| Abe H | 2015 | 2008-2014 | Japan | 64.00 | 236 | 190 | 46 | 80.51 | 7 |
| Ganne-Carrié N | 2000 | 1987-1993 | France | - | 108 | 75 | 33 | 69.44 | 8 |
| Garg SK | 2019 | 2010-2014 | USA | - | 61750 | 37409 | 24341 | 60.58 | 8 |
| Hietanen S | 2021 | 2015-2017 | Finland | 59.06 | 138 | 106 | 32 | 76.81 | 8 |
| Jepsen P | 2016 | 1994-2015 | Denmark | 56.40 | 10650 | 7455 | 3195 | 70.00 | 9 |
| Kalaitzakis E | 2008 | 2004-2006 | Sweden | 58.00 | 87 | 61 | 26 | 70.11 | 7 |
| Kallis C | 2020 | 2014-2018 | UK | 53.40 | 3887 | 2450 | 1437 | 63.03 | 8 |
| Kasztelan-Szczerbińska B | 2015 | - | - | 51.00 | 147 | 107 | 40 | 72.79 | 7 |
| Kim SH | 2016 | 1999-2014 | Japan | 51.50 | 329 | 295 | 34 | 89.67 | 9 |
| Kulkarni K | 2004 | - | USA | - | 89 | 74 | 15 | 83.15 | 6 |
| Lackner C | 2017 | 1995-2009 | Austria | 48.69 | 192 | 135 | 57 | 70.31 | 8 |
| Lin SY | 2019 | 2000-2010 | China(Taiwan) | 46.00 | 1280 | 1147 | 133 | 89.61 | 8 |
| Orrego H | 1987 | - | Canada | 49.89 | 217 | 170 | 47 | 78.34 | 8 |
| Zekanovic D | 2010 | - | Croatia | 58.90 | 60 | 50 | 10 | 83.33 | 6 |
| Yoon EL | 2019 | 2013-2013 | South Korea | 53.00 | 894 | 747 | 147 | 83.56 | 7 |
| Yang TW | 2020 | 2006-2011 | Taiwan | - | 472 | 447 | 25 | 94.70 | 8 |
| Peeraphatdit TB | 2020 | 1999-2017 | USA | 48.00 | 135 | 90 | 45 | 66.67 | 8 |
| Prystupa A | 2021 | - | Poland | 56.23 | 72 | 48 | 24 | 66.67 | 6 |
| Prystupa A | 2016 | - | Poland | 54.90 | 62 | 46 | 16 | 74.19 | 5 |
| Ray G | 2019 | 2011-2016 | India | 38.94 | 395 | - | - | - | 8 |
| Sakamaki A | 2020 | 2006-2019 | Japan | 60.00 | 104 | 83 | 21 | 79.81 | 9 |
| Sandahl TD | 2017 | - | Denmark | 55.88 | 118 | 86 | 32 | 72.88 | 6 |
| Sargenti K | 2015 | 2001-2011 | Sweden | 59.00 | 363 | 255 | 108 | 70.25 | 8 |
| Saunders JB | 1981 | 1959-1976 | UK | - | 242 | - | - | - | 7 |
| Shin S | 2020 | 2003-2016 | South Korea | 59.48 | 949 | 674 | 275 | 71.02 | 9 |
| Solà R | 2006 | 1997-2001 | Spain | 56.00 | 177 | 143 | 34 | 80.79 | 6 |
| Whitfield JB | 2018 | - | Australia/France/Germany/Switzerland/USA | - | 997 | 754 | 243 | 75.63 | 8 |
| Dai, B. | 2013 | 2009-2012 | China(Shandong) | - | 70 | 67 | 3 | 95.71 | 5 |
| Liu, W. S. | 2018 | 2016-2017 | China(Beijing) | 42.70 | 60 | 58 | 2 | 96.67 | 7 |
| Zhang, J. P. | 2005 | 1999-2004 | China(Inner Mongoria) | 43.50 | 158 | 146 | 12 | 92.41 | 8 |
| Rosa H | 2000 | 1986-1998 | Brazil | 46.50 | 201 | 162 | 39 | 80.60 | 8 |
| Adejumo AC | 2020 | 2010-2014 | USA | 46.82 | 21572 | 13162 | 8410 | 61.01 | 8 |
| Nguyen TA | 2016 | 2000-2011 | USA | 48.00 | 6113 | 4208 | 1905 | 68.84 | 8 |
| Deleuran T | 2020 | 1996-2014 | Denmark | 57.00 | 22867 | 15579 | 7288 | 68.13 | 9 |
| Raxitkumar Jinjuvadia | 2015 | 2002-2010 | USA | 53.20 | 326403 | 237621 | 88782 | 72.80 | 9 |
| Roberts SE | 2005 | 1968-1999 | UK | 53.30 | 2802 | 1717 | 1085 | 61.28 | 8 |
| Lee EY | 2015 | 2009-2012 | Czech Republic | 47.50 | 80 | 64 | 16 | 80.00 | 7 |
| Vijayakumar S | 2020 | 2017-2019 | India | 45.13 | 119 | 15 | 104 | 12.61 | 7 |
| Rosen HR | 2016 | - | Europe | 49.00 | 375 | 266 | 109 | 70.93 | 7 |
| Becker U | 1992 | - | - | - | 63 | 34 | 29 | 53.97 | 5 |
| Quintero-Platt G | 2015 | - | Spain | 49.00 | 128 | 113 | 15 | 88.28 | 5 |
| Wei DM | 2021 | 2018-2019 | China(Inner Mongolia) | 51.10 | 79 | 75 | 4 | 94.94 | 8 |
| Armstrong PR | 2022 | 2007-2016 | Ireland | - | 33794 | 23656 | 10138 | 70.00 | 8 |
| Gonzalez HC | 2022 | 2016-2020 | USA | 46.18 | 337 | 156 | 181 | 46.29 | 8 |
| Israelsen M | 2022 | - | UK | 46.00 | 140 | 82 | 58 | 58.57 | 8 |
| Arab JP | 2021 | - | - | 49.00 | 3380 | 2586 | 794 | 76.51 | 6 |
| Tailakh MA | 2021 | - | Israel | 65.82 | 192 | - | - | - | 8 |
| Sullivan MK | 2022 | 2016-2018 | USA | 55.00 | 78 | 45 | 33 | 57.69 | 7 |
| Hagström H | 2022 | 1969-2019 | Sweden | 58.20 | 4028 | 2643 | 1385 | 65.62 | 7 |
| Morales-Arráez D | 2022 | - | Spain/Mexico/South Korea/USA/Colombia/France/Brazil/Chile | 48.00 | 2581 | 1919 | 662 | 74.35 | 8 |
| Laswi H | 2022 | 1998-2018 | USA | 53.48 | 261420 | 192901 | 68519 | 73.79 | 8 |
| Zhang, D.M. | 1995 | - | China(Beijing) | - | 124 | - | - | - | 6 |
| Li, Z. D. | 2012 | 2009-2011 | China(Guangxi) | 53.70 | 132 | 101 | 31 | 76.52 | 8 |
| Zhang, C. J. | 2010 | 2008-2009 | China(Sichuan) | - | 150 | 143 | 7 | 95.33 | 8 |
| Ye, M. C. | 2017 | 2014-2015 | China(Jiangsu) | 55.50 | 155 | 154 | 1 | 99.35 | 8 |
| Li, F. Y. | 2009 | 2004-2005 | China | - | 199 | 192 | 7 | 96.48 | 8 |
| Fu, K. M. | 2019 | 2015-2017 | China(Zhejiang) | 55.20 | 200 | 196 | 4 | 98.00 | 7 |
| Gao, G. F. | 2017 | 2011-2016 | China(Henan) | 49.35 | 206 | 201 | 5 | 97.57 | 7 |
| Zhu, B. | 2015 | 2003-2012 | China(Beijing) | 48.11 | 4132 | 4036 | 96 | 97.68 | 9 |
| Shao, S. | 2018 | 2012-2017 | China(Jilin) | 52.70 | 537 | 521 | 16 | 97.02 | 9 |
| Zhang, Y. Q. | 2009 | - | China(Henan) | 50.00 | 70 | 65 | 5 | 92.86 | 7 |
| Yang, S. M. | 2014 | 2009-2014 | China(Yunnan) | 44.20 | 71 | 66 | 5 | 92.96 | 7 |
| Chen, A. Q. | 2013 | 2011-2012 | China(Guizhou) | 39.80 | 76 | 72 | 4 | 94.74 | 7 |
| Chen, C. Y. | 2008 | 2002-2008 | China(Hubei) | 45.30 | 76 | 74 | 2 | 97.37 | 7 |
| Zhang, M. | 2006 | 1994-2004 | China(Chongqing) | 51.36 | 225 | - | - | - | 9 |
| Liu, C. | 2007 | 2001-2004 | China(Liaoning) | 47.20 | 181 | - | - | - | 8 |
| Liu, L. | 2012 | 2011-2012 | China(Beijing) | 45.00 | 89 | 62 | 27 | 69.66 | 7 |
| Parker R | 2017 | 2009-2014 | Europe and USA | 49.00 | 404 | - | - | - | 7 |
| Sun, S. Q. | 2005 | 1986-1990 | China(InnerMongoria) | 47.00 | 200 | 151 | 49 | 75.50 | 6 |
| Guo, J. | 2003 | - | China(Shandong) | 50.00 | 81 | 72 | 9 | 88.89 | 8 |
| Yang, R. Q. | 2014 | 2003 | China(Xinjiang) | 41.10 | 409 | 371 | 38 | 90.71 | 7 |
| Zhang, Q. D. | 2018 | 2015-2018 | China(Jiangsu) | - | 300 | 281 | 19 | 93.67 | 7 |
| Sun, X. G. | 2001 | 1990-2000 | China(Shandong) | - | 72 | 12 | 60 | 16.67 | 7 |
| Sun, L. Z. | 2010 | 2002-2008 | China(Beijing) | 41.50 | 58 | 6 | 52 | 10.34 | 7 |
| Liu, L. Y. | 2011 | 2008-2009 | China | 52.83 | 466 | 385 | 81 | 82.62 | 8 |
| Liu, Y. | 2009 | 2006-2007 | China | 51.17 | 353 | 288 | 65 | 81.59 | 8 |
| Yang, H. B. | 2012 | 2007-2011 | China(Sichuan) | 55.19 | 74 | 68 | 6 | 91.89 | 6 |
| Li, X. F. | 2020 | 2017-2018 | China(Henan) | 51.03 | 110 | 107 | 3 | 97.27 | 7 |
| Gao, C. | 2014 | 2012-2013 | China(Shanxi) | 49.57 | 120 | 118 | 2 | 98.33 | 8 |
| Liu, C. J. | 2012 | 2005-2011 | China(Shandong) | - | 64 | 61 | 3 | 95.31 | 6 |
| Pang, Y. H. | 2017 | 2014-2016 | China(Qinghai) | 47.00 | 96 | 85 | 11 | 88.54 | 5 |
| Yang, Y. | 2012 | 2009-2011 | China(Xinjiang) | 38.00 | 153 | 111 | 42 | 72.55 | 8 |
| Guo, Z. F. | 2011 | - | China(Shanxi) | 49.00 | 60 | - | - | - | 7 |
| Wu, J. L. | 2003 | 1995-2001 | China(Heilongjiang) | - | 58 | - | - | - | 7 |
| Sun, J. | 2008 | 2007 | China(Jilin) | 43.89 | 152 | - | - | - | 9 |
| Zhang, W. | 2017 | 2014-2016 | China(Jiangsu) | 43.82 | 94 | 53 | 41 | 56.38 | 6 |
| Chen, K. | 2019 | 2016-2018 | China(Tianjin) | 56.36 | 120 | 78 | 42 | 65.00 | 8 |
| Ye, X. F. | 2008 | 2005-2007 | China(Ningxia) | - | 80 | - | - | - | 8 |
| Bai, M. W. M. | 2010 | 2005-2008 | China(Tibet) | 48.10 | 422 | 316 | 106 | 74.88 | 8 |
| Cui, L. | 2010 | 2009-2010 | China(Guizhou) | 48.00 | 186 | 186 | 1 | 99.46 | 8 |
| Wei, J. M. | 2019 | 2016-2018 | China(Henan) | 48.00 | 92 | 77 | 15 | 83.70 | 7 |
| Cao, Z. L. | 2017 | 2013-2016 | China(Guangxi) | 48.50 | 100 | 79 | 21 | 79.00 | 7 |
| Huang, J. Q. | 2011 | 2006-2010 | China(Hainan) | 51.50 | 108 | 99 | 9 | 91.67 | 8 |
| Wang, S. Q. | 2003 | 2000-2003 | China(Henan) | - | 211 | - | - | - | 8 |
| Huang, S. L. | 2005 | - | China(Hunan) | 42.10 | 811 | 781 | 30 | 96.30 | 9 |
| Luo, H. J. | 2001 | - | China(Jiangxi) | 46.00 | 63 | 51 | 12 | 80.95 | 5 |
| Zhao, Y. X. | 2017 | 2011-2014 | China(Gansu) | 46.99 | 1558 | 1546 | 12 | 99.23 | 8 |
| Yuan, Q. Y. | 2011 | 2008-2010 | China(Liaoning) | 36.50 | 368 | - | - | - | 9 |
| Su, J. | 2007 | 2001-2006 | China(Guangdong) | 49.00 | 105 | 103 | 2 | 98.10 | 8 |
| Shu, M. | 2004 | 1999-2003 | China(Liaoning) | 47.00 | 112 | 85 | 27 | 75.89 | 8 |
| Hai, H. | 2007 | 2000-2005 | China(InnerMongoria) | 48.48 | 140 | 130 | 10 | 92.86 | 8 |
| Wang, L. X. | 2004 | 1993-2003 | China(Liaoning) | 48.55 | 164 | 163 | 1 | 99.39 | 8 |
| Wang, L. X. | 2012 | 2000-2010 | China(Liaoning) | 48.55 | 218 | 215 | 3 | 98.62 | 8 |
| Zhao, K. | 2007 | 1996-2006 | China(Jiangxi) | 50.20 | 272 | 256 | 16 | 94.12 | 8 |
| Feng, L. Q. | 2012 | - | China(Hebei) | - | 104 | 98 | 6 | 94.23 | 5 |
| Ye, B. C. | 2005 | 2001-2003 | China(Guangxi) | 38.80 | 58 | 57 | 1 | 98.28 | 6 |
| Zeng, Y. | 2003 | 1995-1998 | China(Shanghai) | 54.80 | 59 | 57 | 2 | 96.61 | 7 |
| Liu, M. Q. | 2010 | 2007-2009 | China(Chongqing) | - | 62 | 56 | 6 | 90.32 | 6 |
| Lai, L. | 2008 | 2004-2007 | China(Xinjiang) | 40.28 | 124 | 93 | 31 | 75.00 | 8 |
| Lu, J. L. | 2011 | 2006-2009 | China(Jiangsu) | 48.50 | 68 | 66 | 2 | 97.06 | 7 |
| Jia, M. S. | 2009 | 2005-2007 | China(Henan) | 52.50 | 70 | 68 | 2 | 97.14 | 6 |
| Liu, C. H. | 2011 | 2008-2011 | China(Jilin) | 45.00 | 78 | 55 | 23 | 70.51 | 5 |
| Hu, H. Y. | 2009 | 2003-2008 | China(Henan) | 48.50 | 85 | 80 | 5 | 94.12 | 5 |
| Yin, H. Y. | 2009 | 2005-2008 | China(Guangxi) | - | 78 | 72 | 6 | 92.31 | 7 |
| Yu, S. H. | 2005 | 2000-2005 | China(Henan) | 43.00 | 430 | 426 | 4 | 99.07 | 6 |
| Xu, F. H. | 2012 | 2008-2011 | China(Shandong) | 45.70 | 263 | 227 | 36 | 86.31 | 7 |
| Chen, H. J. | 2009 | 2005-2008 | China(Guangdong) | 52.00 | 95 | 94 | 1 | 98.95 | 7 |
| Wang, H. Y. | 2009 | 2001-2008 | China(Heilongjiang) | 49.00 | 105 | 103 | 2 | 98.10 | 6 |
| Tan, H. J. | 2006 | 2001-2005 | China(Guangxi) | 46.30 | 78 | 72 | 6 | 92.31 | 7 |
| Liu, J. J. | 2010 | 2001-2007 | China(Shaanxi) | 48.60 | 123 | 116 | 7 | 94.31 | 8 |
| Lu, X. L. | 2004 | - | China(Shaanxi) | - | 83 | 82 | 1 | 98.80 | 8 |
| Ma, J. H. | 2008 | 2003-2007 | China(Henan) | 43.70 | 98 | 93 | 5 | 94.90 | 5 |
| Qiu, Y. H. | 2014 | 2010-2013 | China(Hainan) | 43.40 | 62 | 57 | 5 | 91.94 | 6 |
| Song, Y. Y. | 2011 | 2008-2009 | China(Hunan) | - | 224 | - | - | - | 8 |
| Ren, C. Y. | 2011 | 2008-2010 | China(Heilongjiang) | 41.20 | 72 | 69 | 3 | 95.83 | 5 |
| Liu, Y. | 2004 | 1994-2003 | China(Jilin) | 50.40 | 237 | 235 | 2 | 99.16 | 8 |
| Ma, Y. | 2012 | 2009-2011 | China(Henan) | - | 140 | 92 | 48 | 65.71 | 8 |
| Zhang, J. | 2012 | 2008-2012 | China(Shaanxi) | 60.89 | 167 | 163 | 4 | 97.60 | 8 |
| Chi, B. R. | 2007 | 2000-2004 | China | 51.22 | 902 | 893 | 9 | 99.00 | 7 |
| Wang, D. | 2018 | 2016-2018 | China(Tibet) | 38.50 | 200 | 164 | 36 | 82.00 | 8 |
| Yan, H. | 2007 | 2005 | China(Shaanxi and Gansu) | 38.26 | 58 | 51 | 7 | 87.93 | 7 |
| Jiao, R. B. | 2019 | 2013-2018 | China(Anhui) | 53.01 | 137 | 134 | 3 | 97.81 | 7 |
| Ye, M. C. | 2018 | 2013-2015 | China(Jiangsu) | - | 273 | 265 | 8 | 97.07 | 8 |
| Guo, J. | 2003 | - | China(Shandong) | 50.00 | 81 | 72 | 9 | 88.89 | 8 |
| Lu, X. L. | 2003 | 2000 | China(Shaanxi) | 36.00 | 82 | - | - | - | 8 |
| Ye, G. L. | 2005 | 1999 | China(Zhejiang) | 38.90 | 44 | - | - | - | 7 |
| Zhang, Q. D. | 2018 | 2015-2018 | China(Jiangsu) | - | 300 | 281 | 19 | 93.67 | 7 |
| Li, Q. N. | 2017 | 2016 | China(Shaanxi) | - | 397 | 381 | 16 | 95.97 | 9 |
| Li, X. F. | 2008 | 2005-2006 | China(Hunan) | 44.50 | 46 | 38 | 8 | 82.61 | 7 |
| Lu, G. T. | 2019 | 2017-2018 | China(Guangdong) | 49.35 | 248 | 244 | 4 | 98.39 | 8 |
| Tang, M. | 2011 | 2007-2010 | China(Jilin) | 34.80 | 236 | 230 | 6 | 97.46 | 7 |
| Jiang, N. | 2011 | - | China(Hubei) | 34.80 | 196 | 194 | 2 | 98.98 | 8 |
| Huang, J. A. | 2008 | 2005-2007 | China(Guangxi) | 34.80 | 196 | 194 | 2 | 98.98 | 6 |
| Zheng, C. C. | 2013 | 2006-2010 | China(Guangdong) | 52.90 | 141 | 139 | 2 | 98.58 | 8 |
| Luo, Z. W. | 2018 | 2013-2017 | China(Guangdong) | 48.90 | 70 | 59 | 11 | 84.29 | 6 |
| Yao, J. H. | 2011 | 2010-2011 | China(Yunnan) | 47.89 | 84 | - | - | - | 8 |
| Li, Y. M. | 2003 | 1999-2000 | China(Zhejiang) | 38.80 | 1584 | - | - | - | 8 |
| Pu, G. H. | 2012 | 2009-2011 | China(Jilin) | - | 65 | 60 | 5 | 92.31 | 9 |
| Wu, Y. P. | 2012 | 2008-2009 | China(Yunnan) | 49.00 | 75 | - | - | - | 7 |
| Zhang, Z. D. | 2009 | 2003-2007 | China(Zhejiang) | 30.50 | 40 | - | - | - | 8 |
| Zhao, C. Y. | 2008 | 2003-2007 | China(Hebei) | 50.50 | 286 | - | - | - | 8 |
| Zhou, Z. S. | 2014 | - | China | 47.58 | 4379 | 4278 | 101 | 97.69 | 7 |

**Supplementary Table. 4** Characteristics of included studies for analysis of overall ARLD incidence

| Author | Publication year | Study country | Mean age at baseline (yrs) | Population without ARLD at baseline (N) | Male (n) | New ARLD  (n) | Male | Follow up year | Total follow up  (person. yrs) | Incidence  (per 1000 person.yrs) | Total score  (0-9) |
| --- | --- | --- | --- | --- | --- | --- | --- | --- | --- | --- | --- |
| Saunders JB | 1981 | UK | - | 275000 | - | 242 | 161 | 17.8 | 4882191.78 | 0.050 | 7 |
| Bergman BP | 2015 | UK | - | 172741 | - | 2175 | 2049 | 29.3 | 5061311.30 | 0.430 | 8 |
| Bergman BP | 2015 | UK | - | 56205 | 50970 | 677 | 651 | 29.3 | 1646806.50 | 0.411 | 8 |
| Askgaard G | 2015 | Denmark | - | 55917 | 26696 | 342 | 257 | 14.9 | 833163.30 | 0.410 | 9 |
| Askgaard G | 2021 | Denmark | 58.0 | 5800000 | - | 17473 | 12092 | 10.0 | 58000000.00 | 0.301 | 7 |
| Sandahl TD | 2011 | Denmark | 51.0 | 5200000 | - | 1951 | 1226 | 9.9 | 51287671.23 | 0.038 | 9 |
| Lee JY | 2019 | South Korea | - | 48900000 | - | 39800 | 35552 | 5.0 | 244500000.00 | 0.163 | 7 |
| Hauksson K | 2020 | Iceland | 57.0 | 16000 | 11680 | 159 | 60 | 14.7 | 235397.26 | 0.675 | 7 |

**Supplementary Table. 5** Characteristics of included studies for analysis of overall ARLD Mortality

| Author | Publication Year | Study Country/City | Study Period | Mean age at baseline (yrs) | ARLD  (n) | Male  (n) | Death  (n) | Mortality  (%) | Total score  (0-9) |
| --- | --- | --- | --- | --- | --- | --- | --- | --- | --- |
| Saunders JB | 1981 | UK | 1959-1976 | - | 242 | 0 | 167 | 69.01 | 7 |
| Altamirano J | 2014 | Spain | 2000-2008 | 49.00 | 121 | 78 | 35 | 28.93 | 7 |
| Björnsson ES | 2020 | Iceland | 2001-2016 | 56.00 | 158 | 114 | 11 | 6.96 | 8 |
| Kalaitzakis E | 2008 | Sweden | 2004-2006 | 58.00 | 87 | 61 | 23 | 26.44 | 7 |
| Santos SGRD | 2018 | Brazil | 2009-2014 | 55.60 | 152 | 144 | 22 | 14.47 | 7 |
| Klatsky AL | 1992 | USA | 1978-1985 | 48.50 | 68 | 42 | 40 | 58.82 | 7 |
| Nakamura S | 1979 | Japan | 1972-1977 | - | 130 | 123 | 4 | 3.08 | 7 |
| Beck JI | 2017 | UK | 2006-2010 | 48.00 | 82 | 49 | 55 | 67.07 | 8 |
| Alves PS | 1982 | Portugal | 1966-1978 | - | 463 | 339 | 216 | 46.65 | 7 |
| Ravi S | 2017 | UK | 2004-2012 | 47.99 | 105 | 69 | 56 | 53.33 | 7 |
| Mancebo A | 2013 | Spain | 1992-2010 | 54.10 | 264 | 225 | 62 | 23.48 | 8 |
| Orrego H | 1983 | Canada | - | 49.90 | 253 | 201 | 51 | 20.16 | 8 |
| Goyal SK | 2014 | India | 2011-2012 | 44.81 | 104 | 0 | 32 | 30.77 | 7 |
| Rosa H | 2000 | Brazil | 1986-1998 | 46.50 | 201 | 162 | 25 | 12.44 | 8 |
| Hubbell FA | 1989 | USA | 1980-1983 | 55.20 | 185 | 181 | 57 | 30.81 | 8 |
| Ratib S | 2015 | UK | 1998-2009 | - | 2756 | 1834 | 1452 | 52.69 | 9 |
| Xie YD | 2013 | China(Beijing) | 2000-2011 | 50.48 | 205 | 204 | 14 | 6.83 | 8 |
| Kim SH | 2016 | Japan | 1999-2014 | 51.50 | 329 | 295 | 120 | 36.47 | 9 |
| Barritt AS 4th | 2019 | USA | 2010-2012 | 57.00 | 112495 | 80434 | 7425 | 6.60 | 7 |
| Charni F | 2011 | France | 1995-2005 | 55.00 | 253 | 185 | 94 | 37.15 | 8 |
| Deleuran T | 2012 | Denmark | 1997-2010 | 52.30 | 194 | 99 | 59 | 30.41 | 8 |
| Liangpunsakul S | 2011 | USA | 2007-2007 | 53.20 | 56809 | 41471 | 3881 | 6.83 | 9 |
| Ray G | 2019 | India | 2011-2016 | 38.94 | 395 | 0 | 315 | 79.75 | 8 |
| Božin T | 2021 | Croatia | 2014-2018 | 55.80 | 70 | 52 | 16 | 22.86 | 8 |
| Deleuran T | 2016 | Denmark | 1996-2014 | 57.00 | 22734 | 15686 | 10883 | 47.87 | 9 |
| Vaz K | 2021 | Australia | 2010-2019 | 51.00 | 126 | 72 | 32 | 25.40 | 9 |
| Huang A | 2017 | China(Beijing) | 2002-2013 | - | 7422 | 7281 | 117 | 1.58 | 8 |
| Serra MA | 2003 | Spain | 1973-1997 | 51.00 | 213 | 183 | 49 | 23.00 | 9 |
| Lyra AC | 2020 | Brazil | 2006-2015 | - | 160093 | 130767 | 98070 | 61.26 | 8 |
| Ganne-Carrié N | 2018 | France and Belgian | 2010-2016 | 58.40 | 652 | 440 | 14 | 2.15 | 8 |
| Singal AK | 2012 | USA | 1998-2007 | - | 111726 | 79566 | 3541 | 3.17 | 8 |
| Otete H | 2018 | Denmark | 1997-2014 | 56.00 | 17779 | 2520 | 98 | 0.55 | 8 |
| Dubois M | 2020 | Switzerland | 2010-2017 | 54.00 | 55 | 31 | 24 | 43.64 | 6 |
| Lackner C | 2017 | Austria | 1995-2009 | 48.69 | 192 | 135 | 88 | 45.83 | 8 |
| Singal AK | 2011 | USA | 1993-2008 | 44.86 | 76 | 42 | 13 | 17.11 | 7 |
| Nahon P | 2016 | France | 2000-2010 | 57.80 | 237 | 190 | 100 | 42.19 | 8 |
| Atkinson SR | 2020 | Denmark | - | - | 824 | 520 | 184 | 22.33 | 7 |
| Sandahl TD | 2011 | Denmark | 1999-2008 | 51.00 | 1951 | 1226 | 283 | 14.51 | 9 |
| Nilsson E | 2016 | Sweden | 2001-2010 | 59.50 | 759 | 552 | 565 | 74.44 | 8 |
| Lee JY | 2019 | South Korea | 2008-2012 | - | 39800 | 35552 |  | 0.00 | 9 |
| Sahlman P | 2016 | Finland | 1996-2013 | - | 11871 | 8794 | 8440 | 71.10 | 9 |
| Deltenre P | 2002 | France | 1991-1993 | 49.76 | 89 | 60 | 56 | 62.92 | 6 |
| Ganne-Carrié N | 2000 | France | 1987-1993 | - | 108 | 75 | 60 | 55.56 | 8 |
| Alvarez MA | 2011 | Spain | 1998-2001 | 56.00 | 165 | 135 | 116 | 70.30 | 9 |
| Bergman BP | 2015 | UK | 1981-2012 | 29.30 | 677 | 651 | 445 | 65.73 | 8 |
| Sørensen HT | 2003 | Denmark | 1982-1993 | 56.30 | 6139 | 4353 | 4230 | 68.90 | 9 |
| Dunn W | 2005 | USA | 1995-2001 | 47.00 | 73 | 23 | 16 | 21.92 | 8 |
| Srikureja W | 2005 | USA | 1997-2002 | 43.00 | 202 | 163 | 29 | 14.36 | 8 |
| Thompson JA | 2018 | USA | 2006-2006 | 54.00 | 15496 | 10537 | 7637 | 49.28 | 8 |
| Trimble G | 2013 | USA | 1988-1994 | 41.54 | 148 | 114 | 21 | 14.19 | 7 |
| Fernandes SR | 2017 | USA | 2005-2015 | 51.00 | 170 | 126 | 51 | 30.00 | 9 |
| Becker U | 1996 | Denmark | 1976-1978 | - | 385 | 280 | 98 | 25.45 | 8 |
| Daswani R | 2018 | India | 2015-2017 | 41.00 | 183 | 180 | 80 | 43.72 | 6 |
| Radisavljevic MM | 2017 | - | - | 54.46 | 87 | 79 | 24 | 27.59 | 7 |
| Semb S | 2016 | Denmark | 1976-1987 | 50.40 | 357 | 244 | 307 | 85.99 | 5 |
| Solà R | 2006 | Spain | 1997-2001 | 56.00 | 177 | 143 | 47 | 26.55 | 6 |
| Orrego H | 1987 | Canada | - | 49.89 | 217 | 170 | 80 | 36.87 | 8 |
| Mallet V | 2021 | France | 2020 | 70.00 | 3623 | 0 | 783 | 21.61 | 7 |
| Fujimoto M | 1999 | Japan | 1988-1997 | 53.20 | 105 | 95 | 7 | 6.67 | 5 |
| Kasztelan-Szczerbińska B | 2015 | - | - | 51.00 | 147 | 107 | 12 | 8.16 | 7 |
| Bang UC | 2017 | Denmark | 1995-2014 | 56.01 | 6161 | 3932 | 4089 | 66.37 | 7 |
| Kallis C | 2020 | UK | 2014-2018 | 53.40 | 3887 | 2450 | 534 | 13.74 | 8 |
| Pang JX | 2015 | Canada | 2008-2012 | 49.00 | 122 | 73 | 21 | 17.21 | 9 |
| Jepsen P | 2012 | Denmark | 1993-2005 | - | 8482 | 5655 | 5734 | 67.60 | 9 |
| Al-Azzawi Y | 2020 | USA | - | 47.60 | 196 | 136 | 34 | 17.35 | 5 |
| Nøjgaard C | 2003 | Europe | - | 49.00 | 370 | 277 | 75 | 20.27 | 7 |
| Nault JC | 2013 | France | 2007-2009 | 62.00 | 295 | 253 | 30 | 10.17 | 8 |
| Orntoft NW | 2014 | Denmark | 1999-2008 | - | 1951 | 0 | 1001 | 51.31 | 8 |
| Brunt PW | 1974 | UK | 1960-1967 | 50.12 | 258 | 183 | 72 | 27.91 | 5 |
| Wang X | 2014 | India | 2009-2011 | 45.00 | 722 | 703 | 71 | 9.83 | 8 |
| Heydtmann M | 2013 | UK | 2006-2007 | 50.00 | 124 | 87 | 50 | 40.32 | 6 |
| Silva MJ | 2015 | Portugal | 2003-2012 | 52.98 | 52174 | 0 | 4381 | 8.40 | 8 |
| Altamirano J | 2011 | Mexico | 2006-2008 | 43.00 | 175 | 149 | 89 | 50.86 | 8 |
| Makar M | 2021 | USA | - | 59.40 | 29906 | 24782 | 1015 | 3.39 | 8 |
| Currie C | 2016 | UK | 2009-2010 | 53.89 | 9165 | 6347 | 2145 | 23.40 | 8 |
| Yoon EL | 2019 | South Korea | 2013-2013 | 53.00 | 894 | 747 | 263 | 29.42 | 7 |
| Kulkarni K | 2004 | USA | - | - | 89 | 74 | 24 | 26.97 | 6 |
| Bouttell J | 2016 | UK | 1991-2011 | - | 35208 | 24008 | 6137 | 17.43 | 8 |
| Sandahl TD | 2017 | Denmark | - | 55.88 | 118 | 86 | 50 | 42.37 | 6 |
| Nguyen TA | 2016 | USA | 2000-2011 | 48.00 | 6113 | 4208 | 267 | 4.37 | 8 |
| Hietanen S | 2021 | Finland | 2015-2017 | 59.06 | 138 | 106 | 64 | 46.38 | 8 |
| Roberts SE | 2005 | UK | 1968-1999 | 53.30 | 2802 | 1717 | 775 | 27.66 | 8 |
| Ray G | 2014 | India | 2003-2011 | - | 262 | 0 | 166 | 63.36 | 8 |
| Vijayakumar S | 2020 | India | 2017-2019 | 45.13 | 119 | 15 | 5 | 4.20 | 7 |
| Quintero-Platt G | 2015 | Spain | - | 49.00 | 128 | 113 | 54 | 42.19 | 5 |
| Long, Y. H. | 2006 | China(Beijing) | 2002-2003 | - | 154 | 129 | 20 | 12.99 | 8 |
| Zhang, J. P. | 2005 | China (InnerMongoria) | 1999-2004 | 43.50 | 158 | 146 | 4 | 2.53 | 8 |
| Tao, S. | 2015 | China(Tianjin) | 2011-2014 | - | 211 | - | 11 | 5.21 | 8 |
| Sun, L. Z. | 2010 | China(Beijing) | 2002-2008 | 41.50 | 58 | 58 | 6 | 10.34 | 7 |
| Zhang, Y. Q. | 2009 | China(Henan) | - | 50.00 | 70 | 65 | 5 | 7.14 | 7 |
| Yang, S. M. | 2014 | China(Yunnan) | 2009-2014 | 44.20 | 71 | 66 | 10 | 14.08 | 7 |
| Liu, C. | 2007 | China(Liaoning) | 2001 | 47.20 | 181 | 87 | 1 | 0.55 | 8 |
| Liu, L. | 2012 | China(Beijing) | 2011-2012 | 45.00 | 89 | 62 | 4 | 4.49 | 7 |
| Yang, H. B. | 2012 | China(Sichuan) | 2007-2011 | - | 74 |  | 10 | 13.51 | 6 |
| Su, J. | 2007 | China(Guangdong | 2001-2006 | 49.00 | 105 | 103 | 2 | 1.90 | 8 |
| Zhao, K. | 2007 | China(Jiangxi) | 1996-2006 | 50.20 | 272 | 266 | 16 | 5.88 | 8 |
| Ye, B. C. | 2005 | China(Guangxi) | 2001-2003 | 38.80 | 58 | 57 | 3 | 5.17 | 6 |
| Zeng, Y. | 2003 | China(Shanghai) | 1995-1998 | 54.80 | 59 | 57 | 1 | 1.69 | 7 |
| Liu, M. Q. | 2010 | China(Chongqing) | 2007-2009 | - | 62 | 56 | 2 | 3.23 | 6 |
| Du, G. X. | 2007 | China(Jiangsu) | - | 52.30 | 82 | 60 | 4 | 4.88 | 5 |
| Hu, H. Y. | 2009 | China(Henan) | 2003-2008 | 48.50 | 85 | 80 | 5 | 5.88 | 5 |
| Wang, H. Y. | 2009 | China (Heilongjiang) | 2001-2008 | 49.00 | 105 | 103 | 2 | 1.90 | 6 |
| Ma, J. H. | 2008 | China(Henan | 2003-2007 | 43.70 | 98 | 93 | 3 | 3.06 | 5 |
| Dai, B. | 2013 | China(Shandong) | 2009-2012 | - | 70 | 67 | 4 | 5.71 | 5 |
| Ren, C. Y. | 2011 | China (Heilongjiang) | 2008-2010 | 41.20 | 72 | 69 | 3 | 4.17 | 5 |
| Yang, Y. | 2005 | China(Henan) | 2000-2005 | - | 72 | 72 | 1 | 1.39 | 7 |
| Liu, Y. | 2004 | China(Jilin) | 1994-2003 | 50.40 | 237 | 235 | 31 | 13.08 | 8 |
| Sun, X. G. | 2001 | China(Shandong) | 1990-2000 | - | 72 | 72 | 12 | 16.67 | 7 |
| Ma, Y. | 2012 | China(Henan | 2009-2011 | 60.89 | 167 | 163 | 4 | 2.40 | 8 |
| Liu, W. S. | 2018 | China(Beijing) | 2016-2017 | 42.70 | 60 | 58 | 11 | 18.33 | 7 |
| Zhou, Z. S. | 2014 | China(Beijing) | 2002-2011 | 47.58 | 4379 | 4278 | 1013 | 23.13 | 7 |
| Armstrong PR | 2022 | Ireland | 2007-2016 | - | 33794 | 23656 | 2880 | 8.52 | 8 |
| Israelsen M | 2022 | UK | - | 46.00 | 140 | 82 | 67 | 47.86 | 8 |
| Sullivan MK | 2022 | USA | 2016-2018 | 55.00 | 78 | 45 | 37 | 47.44 | 7 |
| Hagström H | 2022 | Sweden | 1969-2019 | 58.20 | 4028 | 2643 | 1779 | 44.17 | 7 |

**Supplementary Figure. 1 The forest plots of global prevalence**

**
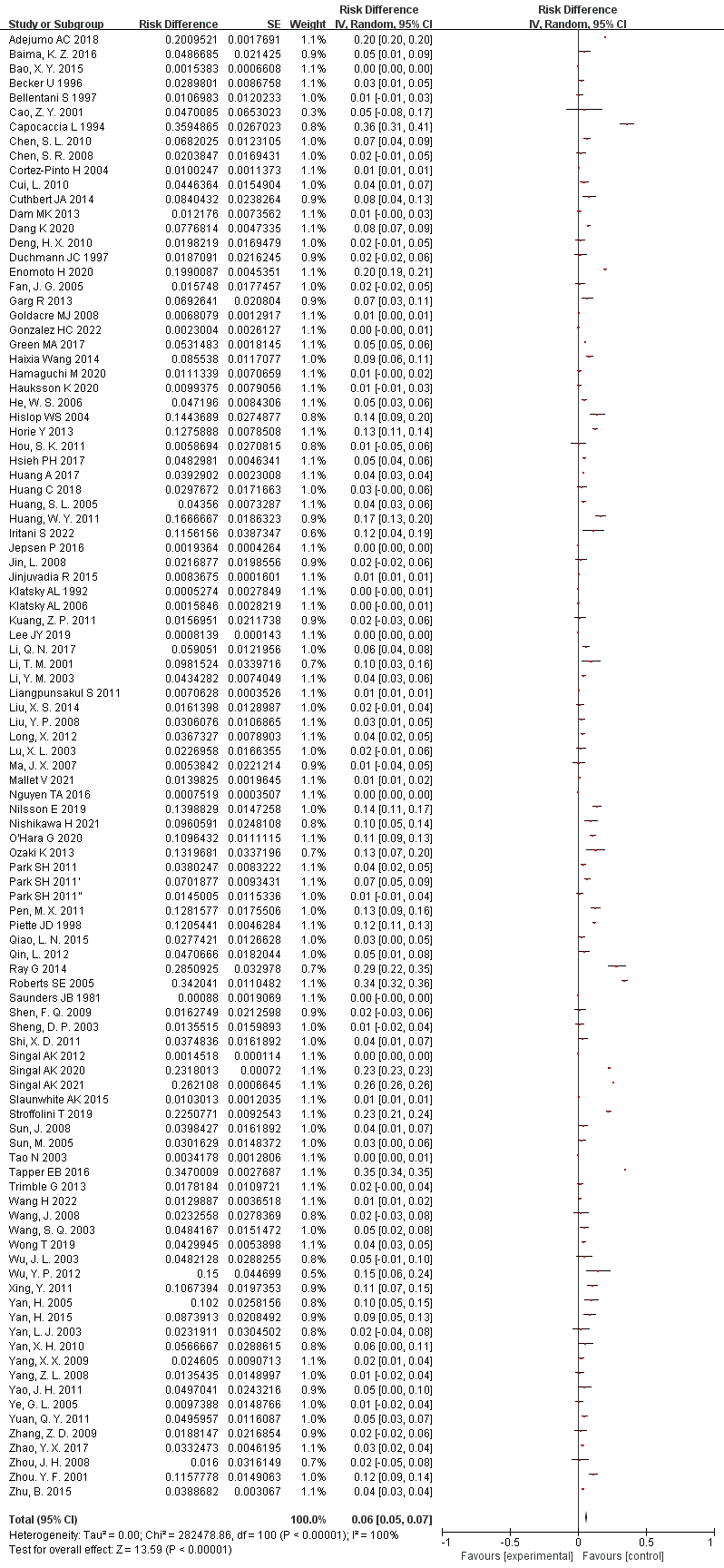
**

**Supplementary Figure. 2 The forest plots of China prevalence**

**
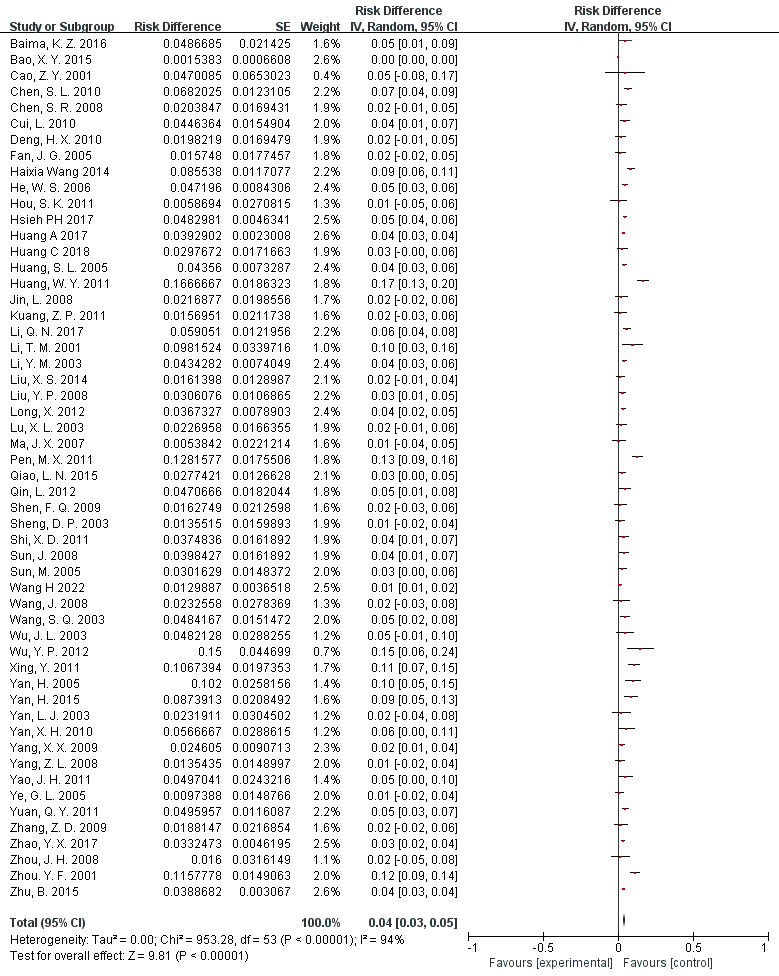
**

**Supplementary Figure. 3 The forest plots of men prevalence**

**
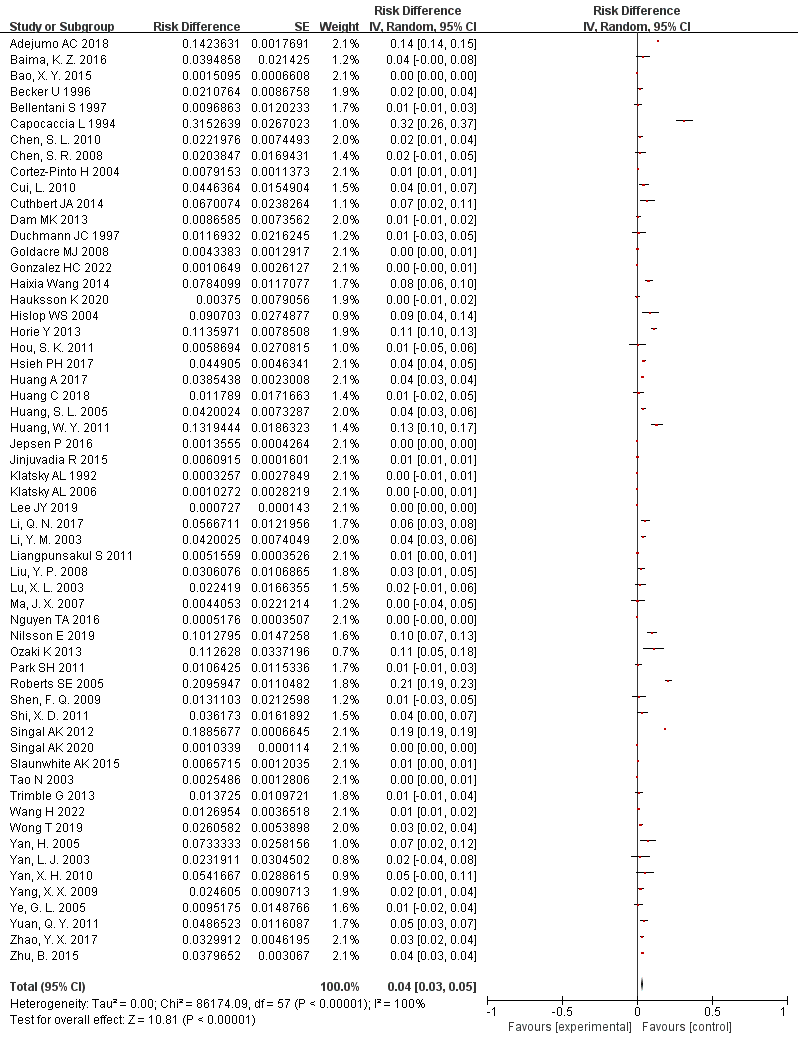
**

**Supplementary Figure. 4 The forest plots of female prevalence**

**
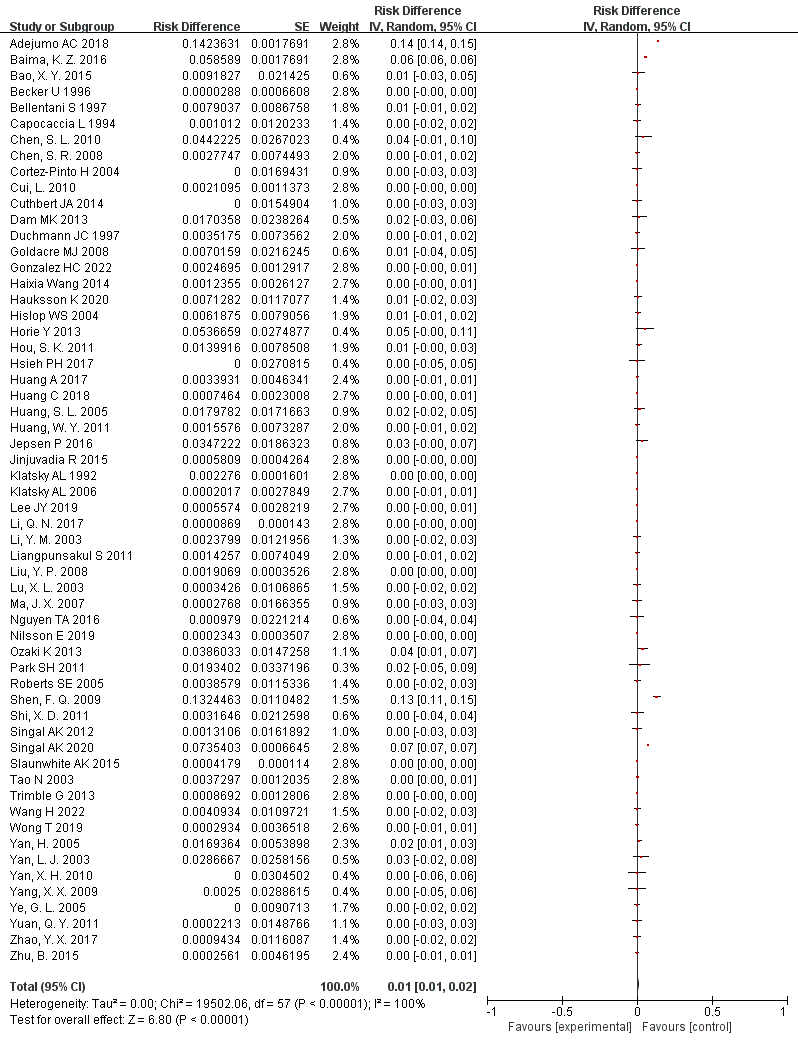
**

**Supplementary Figure.5 Funnel plot of studies included for analysis of overall ARLD prevalence**


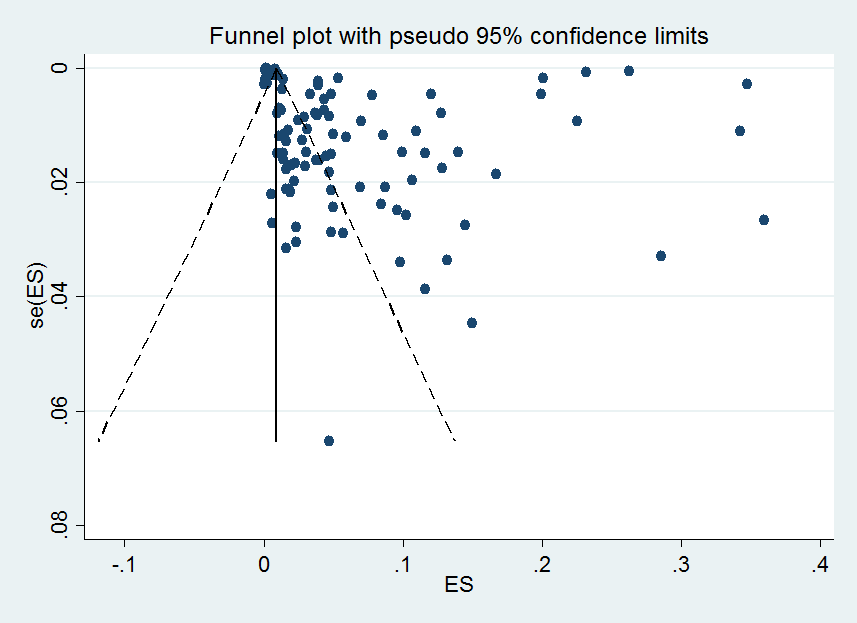

Supplement: Supplementary file 1 — Additional file 1: Supplementary methods. Supplementary Table 1. Search strategy. Supplementary Table 2. Characteristics of included studies for analysis of overall ARLD prevalence. Supplementary Table 3. Summary of articles used to analyze the characteristics of ARLD. Supplementary Table 4. Characteristics of included studies for analysis of overall ARLD incidence. Supplementary Table 5. Characteristics of included studies for analysis of overall ARLD Mortality. Supplementary Figure 1. The forest plots of global prevalence. Supplementary Figure 2. The forest plots of China prevalence. Supplementary Figure 3. The forest plots of men prevalence. Supplementary Figure 4. The forest plots of female prevalence. Supplementary Figure 5. Funnel plot of studies included for analysis of overall ARLD prevalence. [file 12889_2023_15749_MOESM1_ESM.docx]
